# Effects of different types of deep brain stimulation on gait disorders in patients with Parkinson’s disease: a network meta-analysis of randomized controlled trials

**DOI:** 10.3389/fnagi.2025.1723706

**Published:** 2026-01-22

**Authors:** Yunpeng Guo, Zhanyi Zhang, Yixian Hou

**Affiliations:** 1 School of Physical Education, Henan Normal University, Xinxiang, China; 2 Center for General Education Teaching and Research, Northern Henan Medical University, Xinxiang, China

**Keywords:** deep brain stimulation, freezing of gait, gait disorders, gait variability, Parkinson’s disease

## Abstract

**Objective:**

To systematically compare and rank the relative efficacy of different types of deep brain stimulation (DBS) for gait disorders in patients with Parkinson’s disease (PD), with particular emphasis on three outcomes: motor function (MDS-UPDRS III), Freezing of Gait Questionnaire (FOG-Q), and normal gait velocity (cm/s). The goal is to provide evidence-based guidance for individualized neuromodulation strategies.

**Methods:**

Following a preregistered protocol (PROSPERO CRD420251074368), a comprehensive literature search was conducted from January 1, 2000, to June 15, 2025, across PubMed, Embase, Cochrane Library, and CNKI. Randomized controlled trials (RCTs) meeting predefined PICOS criteria were included, and continuous outcome data for MDS-UPDRS III, FOG-Q and normal gait velocity in the Off-Dopa state were extracted. A frequentist network meta-analysis framework (R, netmeta package) was employed, with mean difference (MD) and 95% confidence intervals (CI) as effect estimates. Heterogeneity was quantified using *τ*^2^ and *I*^2^ statistics. Global and local inconsistency tests were applied, and treatment ranking was performed using P-scores. Robustness was examined through fixed- vs. random-effects comparisons, leave-one-out sensitivity analyses, and, where feasible, meta-regression and subgroup analyses.

**Results:**

Twenty-five RCTs were included, involving 324 patients in intervention groups and 324 in control groups. (1) MDS-UPDRS III: Network meta-analysis showed that Low-PPNa-DBS produced the greatest improvement in motor function (MD = −31.20, 95% CI –53.25 to −9.15, *p* = 0.0055), followed by PPN-DBS (MD = −26.00, 95% CI –38.92 to −13.08, *p* ≤ 0.0001) and CuN-DBS (MD = −23.00, 95% CI –42.65 to −3.35, *p* = 0.0218). Additional significant but moderate effects were observed for Posterior-STN-dDBS (MD = −16.55, 95% CI –28.43 to −4.68, *p* = 0.0063), IL-IL-DBS, 60 Hz-STN-DBS, 80 Hz-STN-DBS, STN-DBS, STN + SNr HF-DBS, and STN + SNr LF-DBS. Overall heterogeneity was moderate to high (*τ*^2^ = 12.1151, *I*^2^ = 68.7%), and heterogeneity testing indicated significant heterogeneity (*Q* total = 14.48, df = 5, *p* = 0.0128). (2) FOG-Q: None of the DBS modalities yielded statistically significant improvements in FOG-Q scores (all 95% CIs crossed zero, *p* > 0.05). This outcome exhibited high heterogeneity (*τ*^2^ = 1.3074, *I*^2^ = 88.3%) and significant inconsistency (*Q* total = 25.61, *p* < 0.0001), suggesting that current evidence is insufficient to confirm a definitive DBS benefit for freezing of gait. (3) Gait velocity (cm/s): Network analysis demonstrated that spDBS [MD = 18.06, 95% CI (10.42, 25.70), *p* < 0.0001] and STN-DBS [MD = 17.58, 95% CI (13.20, 21.95), *p* < 0.0001] provided the most pronounced improvements. Conventional aDBS [MD = 14.80, 95% CI (7.71, 21.89)] and GPi-DBS [MD = 13.91, 95% CI (2.40, 25.42)] were associated with moderate benefits. Heterogeneity for this outcome was moderate to high (*τ*^2^ = 12.1151, *I*^2^ = 65.5%), indicating persistent between-study variability.

**Conclusion:**

This network meta-analysis systematically compared the effects of different DBS modalities on gait disorders in PD. For MDS-UPDRS Part III, Low-PPNa-DBS, PPN-DBS, and CuN-DBS yielded the most pronounced and statistically robust improvements. In contrast, analysis of FOG-Q scores revealed no statistically significant effects for any DBS intervention. Regarding gait speed, spDBS and STN-DBS showed clear and significant enhancements.

**Systematic review registration:**

https://www.crd.york.ac.uk/PROSPERO/view/CRD420251074368, Identifier: CRD420251074368.

## Graphical abstract

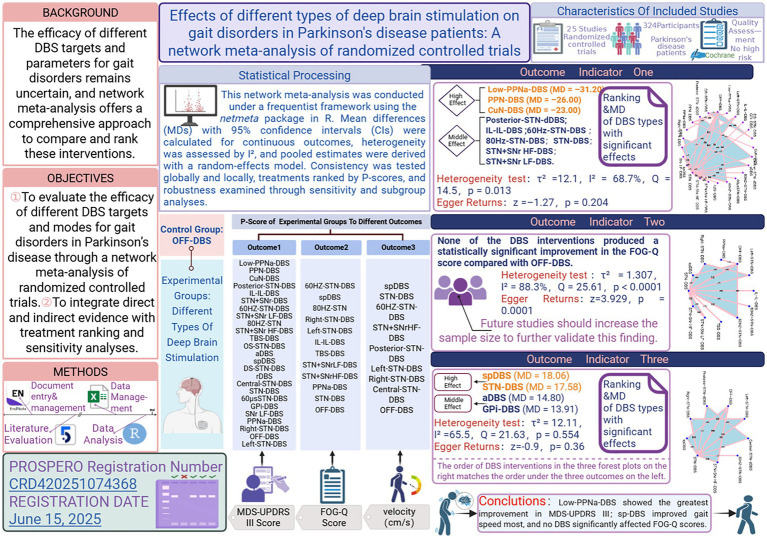

## 1 Introduction

Parkinson’s disease (PD) is a common progressive neurodegenerative disorder worldwide, characterized by core motor symptoms including bradykinesia, rigidity, and resting tremor (Lees et al., 2009). Among these, gait disorders represent one of the most challenging manifestations to manage in the mid-to-late stages of the disease. These gait disturbances substantially reduce functional mobility and independence and are strongly associated with increased fall risk, progressive disability, and heightened caregiving burden (Bloem et al., 2021; Tolosa et al., 2021).

In Parkinson’s disease, gait disorders are an important component of motor dysfunction. Motor function in PD is typically evaluated through motor signs, mobility and balance performance, and quantitative gait parameters (Yin et al., 2025). Gait disorder in PD is broadly characterized as a multi-dimensional locomotor impairment resulting not only from basal ganglia dysfunction but also from degenerative or dysregulated processes involving extra–basal ganglia structures—including the brainstem, cerebellum, cortical regions, and multimodal sensory integration systems—which collectively compromise the automaticity, rhythmicity, and adaptability of walking (Gao et al., 2020; Jankovic, 2008). Clinically, PD-related gait disorder manifests through phenomena such as shuffling steps, reduced stride length, diminished gait speed, increased step-to-step variability, freezing of gait (FOG), and impaired postural control (Mirelman et al., 2019). From a quantitative standpoint, these manifestations can be operationalized through specific spatiotemporal gait metrics, including stride length, gait speed, cadence, swing and stance phase proportions, step variability indices, and turning performance, each of which reflects distinct pathophysiological substrates of the disease (Guo et al., 2022; Nutt et al., 2011; Johansson et al., 2023).

Although pharmacological therapies—such as levodopa (Turcano et al., 2018), dopamine receptor agonists (Perez-Lloret and Rascol, 2010), amantadine (Inzelberg et al., 2006), and catechol-O-methyltransferase (COMT) inhibitors (Fabbri et al., 2022)—can alleviate symptoms during the early stages, disease progression is typically accompanied by motor fluctuations and dyskinesias (Aradi and Hauser, 2020; Cenci et al., 2022; Rascol et al., 2003). These treatments provide only limited benefit for gait disorders, and some agents may induce addictive behaviors or mood disturbances (Debove et al., 2024; Zhang et al., 2016), emphasizing the potential clinical utility of other therapeutic options.

Deep brain stimulation (DBS) has emerged as a major surgical intervention for managing motor complications in advanced PD. DBS can be tailored to individual symptom profiles and targets distinct nodes within the basal ganglia–thalamocortical circuits that mediate diverse PD manifestations (Hariz and Blomstedt, 2022). The subthalamic nucleus (STN-DBS) (Onder and Comoglu, 2024) and the internal segment of the globus pallidus (GPi-DBS) (Vitek, 2002) remain the most widely applied targets. In recent years, technological advances such as adaptive DBS (aDBS) (Beudel and Brown, 2016), device-assisted neuromodulation therapies (Rajan et al., 2022), directional electrodes, and personalized stimulation strategies (Schroter et al., 2024; Yin et al., 2021) have been developed, aiming to achieve more precise modulation of abnormal neural activity through closed- or semi-closed-loop systems. However, the efficacy of different DBS targets and stimulation parameters for gait disorders remains controversial (Cury et al., 2022), and evidence regarding invasive interventions for PD is inconsistent (Deuschl et al., 2022), highlighting the need for more systematic comparative evaluations.

Existing studies on STN-DBS and GPi-DBS largely address overall motor symptoms and are limited by small samples, heterogeneous designs, and reliance on pairwise analyses, leaving the comparative effects of different DBS modalities on gait disorders insufficiently defined (Deuschl et al., 2022; Fan et al., 2021; Lin et al., 2021). Evidence for emerging stimulation paradigms is particularly sparse. Network meta-analysis (NMA) (Hoaglin et al., 2011), by integrating direct and indirect comparisons, enables simultaneous evaluation and ranking of multiple DBS targets and paradigms, thus overcoming the lack of head-to-head trials and providing a more comprehensive framework for gait-specific assessment. Using validated gait-related measures, the present study applies an NMA to compare the efficacy of established and emerging DBS strategies—including DBS-off or sham controls—while examining heterogeneity through subgroup and sensitivity analyses.

## 2 Materials and methods

### 2.1 Study design

This study was conducted as a network meta-analysis aimed at systematically evaluating the effects of different types of deep brain stimulation (DBS) on gait disorders in patients with Parkinson’s disease (PD). The study population consisted of patients with clinically confirmed PD presenting with gait impairments, and only randomized controlled trials (RCTs) were included, and these studies required clear outcome data. The study was designed and implemented in strict accordance with the Preferred Reporting Items for Systematic Reviews and Meta-Analyses (PRISMA) guidelines (Shamseer et al., 2015). Based on the principles of population, intervention, comparator, outcomes, and study design (PICOS) (Methley et al., 2014), a set of predefined inclusion and exclusion criteria was established to determine study eligibility. The protocol was registered on June 15, 2025, in the PROSPERO database (https://www.crd.york.ac.uk/prospero/; registration ID: CRD420251074368) to ensure methodological transparency and reproducibility.

### 2.2 Eligibility criteria based on PICOS

#### 2.2.1 Population

*Included*: Adult patients (≥18 years) diagnosed with idiopathic Parkinson’s disease (PD) according to established diagnostic criteria, presenting clinically relevant gait disturbances that impair mobility. Gait disturbances may manifest as reduced gait speed, shortened step length, increased stride-to-stride variability, freezing of gait (FOG), or other observable walking dysfunctions.

*Excluded*: Patients with secondary or atypical parkinsonism [e.g., multiple system atrophy (MSA), progressive supranuclear palsy (PSP)] or those undergoing surgical interventions other than DBS.

#### 2.2.2 Intervention

*Included*: Various types of DBS targeting key brain regions such as the subthalamic nucleus (STN-DBS) and globus pallidus internus (GPi-DBS), as well as advanced modalities including adaptive DBS (aDBS). Stimulation parameters (site, frequency, mode) were analyzed to explore their effects on gait.

*Excluded*: Non-DBS neuromodulation techniques (e.g., transcranial magnetic stimulation, transcranial direct current stimulation, spinal cord stimulation) or non-neuromodulatory interventions.

#### 2.2.3 Comparator

*Included*: Other DBS types, sham stimulation, off DBS. Head-to-head comparisons of different DBS targets (e.g., STN vs. GPi, STN vs. aDBS, GPi vs. PPN) were eligible, allowing evaluation of differential effects on gait outcomes.

#### 2.2.4 Outcomes

*Primary outcomes*: Quantitative gait-related measures, including MDS-UPDRS Part III (motor examination), gait speed, stride time variability, and Freezing of Gait Questionnaire (FOG-Q) scores. Only studies reporting clearly defined DBS interventions with these measurable gait outcomes were included. Furthermore, eligible studies were restricted to randomized controlled trials (RCTs), including crossover designs, that explicitly reported blinded assessments.

#### 2.2.5 Study design & context

Eligible studies were restricted to randomized controlled trials (RCTs), including crossover designs, that explicitly reported blinded assessments (Savovic et al., 2012). Studies conducted in hospital-based neurology or neurosurgery departments or movement disorder centers were considered. Observational studies, case reports, narrative reviews, and studies without full-text availability were excluded.

### 2.3 Literature search strategy

Because early studies may have methodological limitations in sample size estimation and outcome measurement, the search period was set from January 1, 2000, to June 15, 2025, to enhance the accuracy and credibility of this network meta-analysis. Database search strategies are provided in Supplementary Table S3. The databases searched included PubMed, Embase, Cochrane Library, and CNKI.

Our stringent search and criteria were essential for maintaining internal validity by reducing methodological heterogeneity, though such rigor may inevitably restrict the generalizability of the findings (Turner et al., 2012).

### 2.4 Inclusion and exclusion criteria

#### 2.4.1 Inclusion criteria

(1) Participants: Adult patients (≥18 years) with a confirmed diagnosis of idiopathic PD, regardless of sex, disease duration, or stage, with clinical manifestations of gait impairment (e.g., reduced gait velocity, shortened step length, freezing of gait [FOG], or other gait-related dysfunctions). (2) Intervention: DBS treatment, without restriction on target site or stimulation paradigm. (3) Comparator: Other DBS modalities, DBS-off, or sham stimulation. (4) Study design: RCTs, including parallel-group and crossover designs. Crossover trials were eligible if random allocation to intervention sequences was explicitly described. No restriction was imposed on the duration of the washout period; however, sensitivity analyses were conducted to assess the potential impact of carryover effects in crossover trials at risk, ensuring the robustness of the results. (5) Outcomes: Studies were required to report at least one extractable quantitative gait-related outcome (gait velocity, FOG-Q score, or MDS-UPDRS III score) along with corresponding effect size and variance estimates (standard error or confidence interval).

#### 2.4.2 Exclusion criteria

(1) Patients with secondary or atypical parkinsonism [e.g., multiple system atrophy (MSA), progressive supranuclear palsy (PSP)] or patients undergoing surgical interventions other than DBS. Because such procedures may have distinct mechanisms, targets, and clinical effects, which could introduce heterogeneity and confound the evaluation of DBS-specific outcomes. (2) Non-PD populations or mixed cohorts where PD-specific data could not be extracted. (3) Interventions not involving DBS [e.g., pharmacological therapy alone, rehabilitation, or other neuromodulation techniques such as transcranial magnetic stimulation (TMS), transcranial direct current stimulation (tDCS), or spinal cord stimulation (SCS)]. Studies using TMS, tDCS, or other non-invasive neuromodulation techniques were excluded because these interventions differ fundamentally from DBS with respect to invasiveness, target sites, mechanisms of action, and clinical effects. Including such studies could introduce heterogeneity and reduce the specificity of the analysis. (4) Studies not reporting gait-related outcomes. (5) Non-RCT study designs (e.g., observational studies, case series, case reports, reviews, or expert opinions). (6) Incomplete or unextractable data, with no supplementary information available after contacting the authors. (7) Duplicate publications or repeated use of the same cohort (only the study with the largest sample size or highest methodological quality was retained) (8) Animal or *in vitro* studies.

### 2.5 Data extraction

Search results from each database were imported into EndNote 20 (Clarivate Analytics, Philadelphia, PA, United States) for reference management and duplicate removal. Extracted study characteristics included first author, publication year, intervention type, sample sizes of intervention and control groups, and gait-related outcomes with effect size data. Quality assessment of the included studies was performed using Review Manager 5.4.1 (The Cochrane Collaboration, London, United Kingdom). Two independent reviewers (YG and YH) used customized Excel worksheets to extract data, including author, country, demographic characteristics, and stimulation type. Extraction consistency was assessed by calculating the Pearson correlation coefficient (*r*) across variables (Stoll et al., 2019); the initial overall mean r was 0.81, indicating high reliability. Discrepancies were resolved by discussion, with arbitration by a third reviewer (ZZ) when necessary. Studies for which the requisite outcome data could not be extracted, and for which relevant information could not be obtained from the original authors despite attempts to contact them, were excluded from the analysis. If unsuccessful, data were extracted using WebPlotDigitizer v4.7.^1^ Final analyses were conducted using the effect-size data extracted or derived from each study, including outcome means, measures of variability (e.g., standard deviations or standard errors), and sample sizes, following standard procedures for network meta-analysis (Sutton et al., 1999; Baird, 2018).

To assess the potential influence of carryover effects in crossover trials, we conducted a structured sensitivity analysis focusing on studies with unreported or insufficient washout periods (Mathur and VanderWeele, 2020; Mathur, 2024). All crossover studies lacking explicit washout information or reporting durations considered clinically inadequate (e.g., <24 h) were temporarily excluded, after which the network meta-analysis was re-estimated to evaluate changes in treatment ranking and effect-size estimates relative to the primary analysis. Additionally, crossover trials were stratified into those with reported versus unreported washout periods, and a node-splitting approach was applied to detect any subgroup-specific inconsistencies. This combined strategy allowed us to identify potential hidden carryover effects and ensured the robustness of the overall treatment estimates. When only the mean and range are reported, the standard deviation was estimated according to the method described by Hozo et al. (2005).

Gait-speed values reported in different units (e.g., m/s, cm/s) were manually converted to a uniform scale (cm/s) before analysis, enabling the use of mean differences (MDs) rather than standardized mean differences (SMDs). MDS-UPDRS Part III scores were assessed using standardized and widely adopted scoring systems, ensuring consistency across studies. Therefore, no additional standardization of effect sizes was required, and each outcome was synthesized using either mean differences (MDs) or the original scale, preserving clinical interpretability. The FOG-Q and NFOG-Q questionnaires were used, the outcome measure for FOG-Q was analyzed using the standardized mean difference (SMD) (Kwon and Reis, 2015; Weir et al., 2018), the conversion formulas are provided in Supplementary Note S2. To enhance data comparability and ensure consistency in pooled analyses, all extracted means and standard deviations were uniformly retained to one decimal place using the rounding method, ensuring the robustness. The corresponding definitions are shown in Table 1.

**Table 1:** Primary gait-related outcomes and their scientific rationale.

Gait function domain	Corresponding Outcome	Explanation
Global motor function	MDS-UPDRS Part III	The gold-standard clinician-rated assessment of overall motor impairment in PD, encompassing bradykinesia, rigidity, tremor, and gait/postural components. Provides a comprehensive, validated measure to contextualize gait-specific outcomes and support interpretation of DBS effects across multiple motor domains (Goetz et al., 2008; Evers et al., 2019)
Freezing of gait/advanced motor dysfunction	FOG-Q	A validated, patient-reported questionnaire assessing the severity and frequency of freezing episodes, which are disabling and highly specific to PD gait pathology. Captures subjective motor experiences complementary to objective gait metrics (Thanvi and Treadwell, 2010; Giladi et al., 2000; Mancini et al., 2019)
Gait speed/locomotor control	Gait speed	The most direct and widely reported objective measure of walking performance, reflecting overall gait efficiency, step execution, and motor output. High clinical relevance in Parkinson’s disease (PD) and commonly used in RCTs evaluating DBS interventions (Kirk et al., 2025; Fukuchi et al., 2019)

For studies reporting stride length and cadence instead of gait speed, gait speed was calculated using the following formula (Youdas et al., 2006):
Gait speed(cm/s)=Stride length(cm)×Cadence(steps/s)

This standardization ensures consistency across studies and allows for meaningful synthesis of gait outcomes in the network meta-analysis.

### 2.6 Assessment of risk of bias and methodological quality

This study employed a frequentist framework-based network meta-analysis, with all statistical analyses performed using the network-meta package in R (Daly and Soobiah, 2022). Data integration and management were conducted with Microsoft Excel software (Microsoft Corporation, Redmond, WA, United States). For all study outcomes, mean difference (MD) with corresponding 95% confidence intervals (CIs) was used as the effect size. Overall heterogeneity was assessed by the *I*^2^ statistic (Ades et al., 2024), categorized as low (0–40%), moderate (40–70%), or high (70–100%). Initially, a network evidence plot was generated to illustrate the comparative relationships among interventions. A fixed-effects model was applied to pool effect sizes when heterogeneity was low (0–40%); otherwise, a random-effects model was used., and both global and local inconsistency (node-splitting method) tests were performed to evaluate agreement between direct and indirect evidence. When significant inconsistency was detected (*p* < 0.10), finally, the relative efficacy of interventions was ranked using P-score (Rucker and Schwarzer, 2015).

To systematically evaluate the robustness of the network meta-analysis results, a multi-level sensitivity analysis strategy was implemented. First, results obtained from random-effects and fixed-effect models were compared to assess the potential impact of model selection on effect estimation (Rucker et al., 2020; Lin et al., 2017), the fixed-effect model assumes a common true effect across studies and estimates pooled effects purely based on within-study variance. Consistency between fixed- and random-effects estimates was taken as evidence of robustness to model assumptions. Second, analyses were repeated after excluding studies at high risk of bias to minimize the influence of study quality. Third, leave-one-out sensitivity analysis was conducted by sequentially omitting each individual study to examine its influence on the overall effect estimate. In addition, potential sources of heterogeneity were explored through meta-regression and prespecified subgroup analyses (Venekamp et al., 2014), stratified by clinical characteristics such as mean disease duration, mean age, and washout period, thereby providing a scientific basis for interpreting the findings. A *p*-value <0.05 was considered statistically significant, <0.01 as highly significant, and <0.001 as extremely significant.

## 3 Results

### 3.1 Search results, basic characteristics, and quality assessment

**Figure 1:**
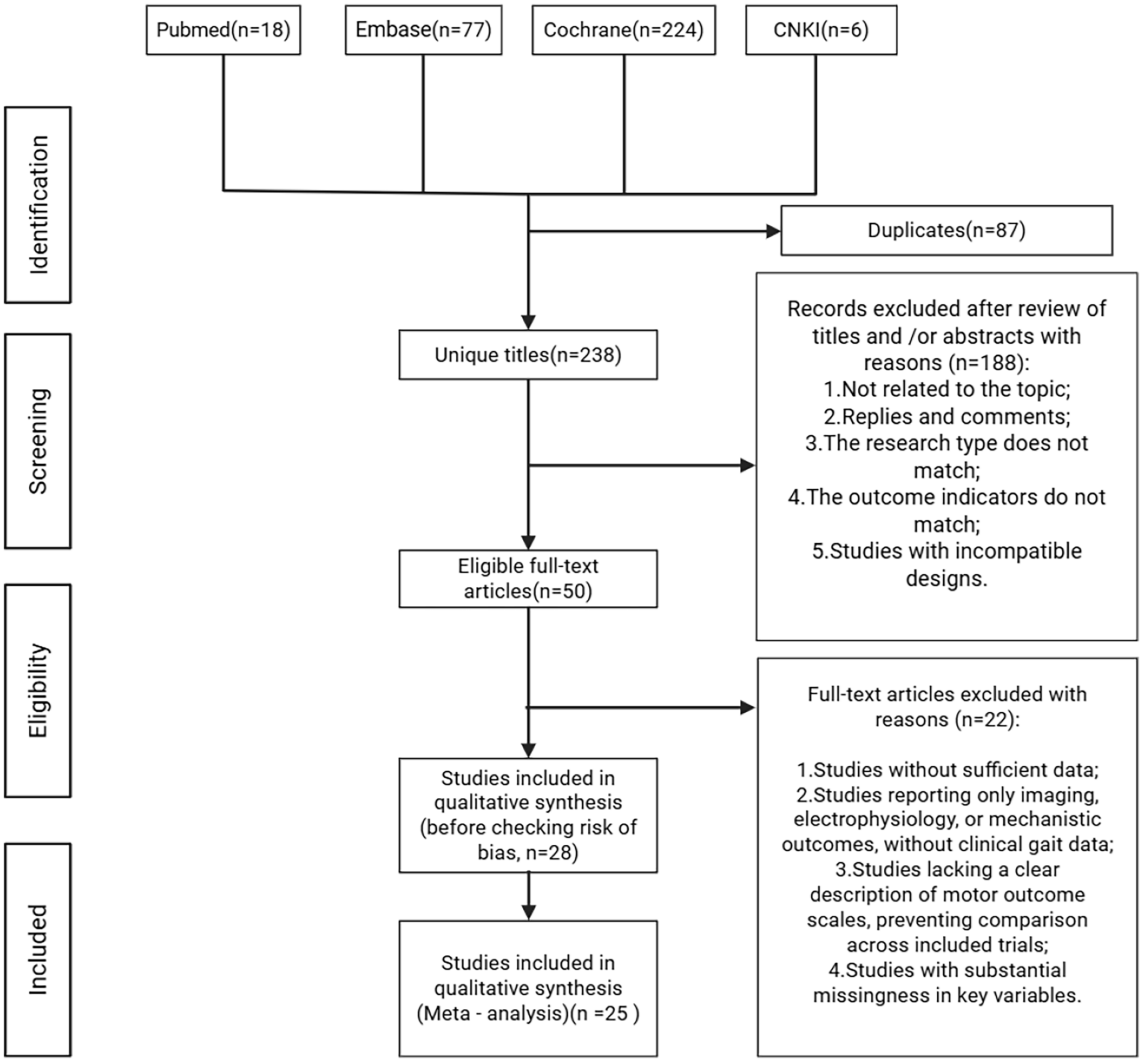
PRISMA flow chart of study inclusion and exclusion.

A total of 325 records were identified through the systematic search. After import into EndNote 20, 87 duplicates were removed. Title and abstract screening were then performed to exclude records unrelated to the study topic, animal studies, reviews, and prior meta-analyses, leaving 50 articles for full-text assessment. Studies for which complete outcome data could not be obtained were excluded. After careful full-text review, 25 studies met the prespecified inclusion criteria. The study selection flow is presented in Figure 1, and detailed characteristics of the included studies are provided in the Supplementary Table S1.

From initial retrieval to final study selection, all exclusion criteria were applied using refined and standardized procedures. In addition to the key exclusion categories presented in the PRISMA flowchart (Page et al., 2021), a detailed list of all specific exclusion reasons is provided in the Supplementary material for full transparency.

**Figure 2:**
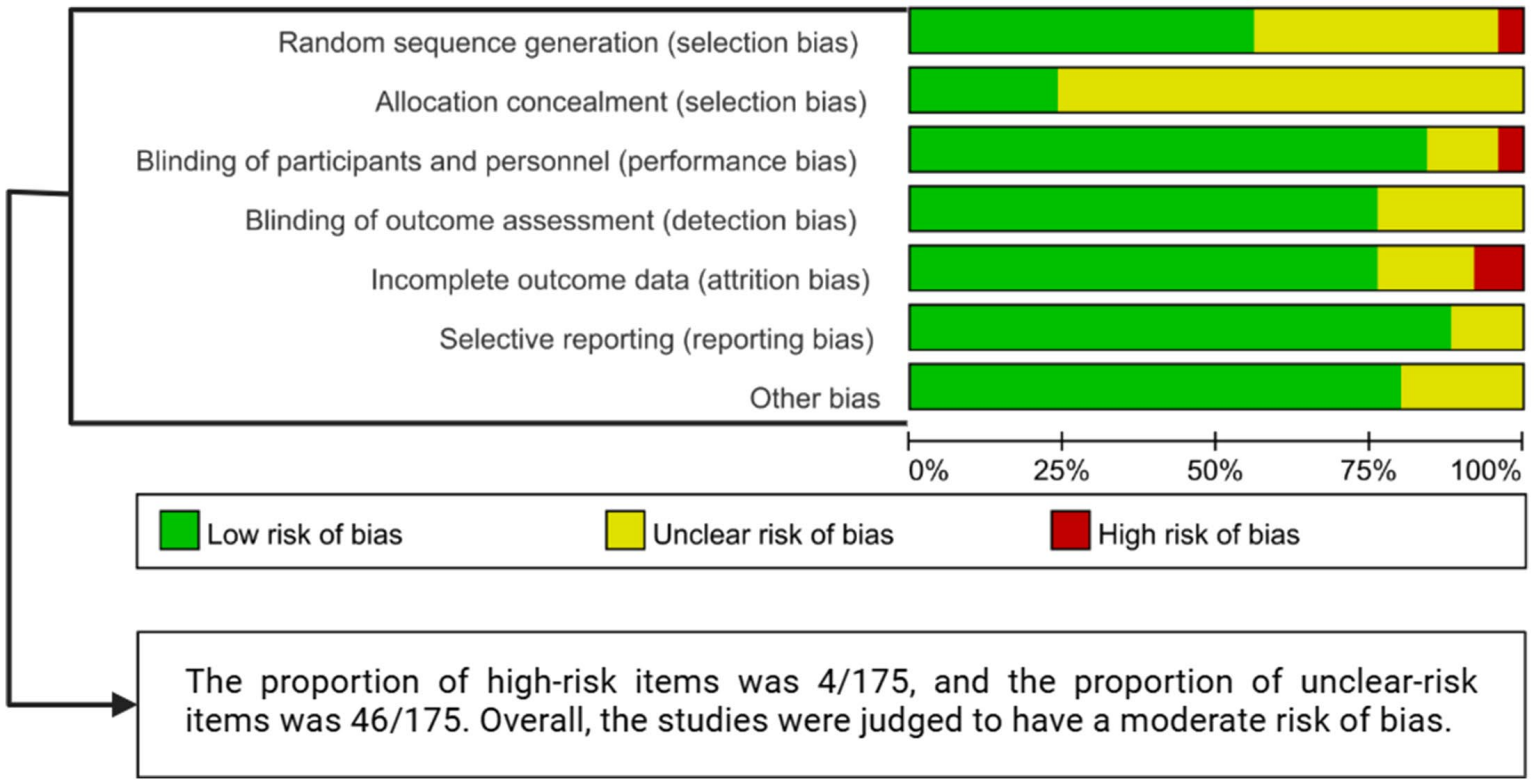
Quality assessment of included literature.

The basic characteristics and serial numbers of the included studies are presented in Supplementary Table S1. Among the 25 included trials, 11 were published before 2020 [studies 6 and 7 (Varriale et al., 2018; Fasano et al., 2011), 12–15 (Xie et al., 2018; Weiss et al., 2013; Nosko et al., 2015; Welter et al., 2015), 18 and 19 (Lizarraga et al., 2016; Petry-Schmelzer et al., 2022), 21 and 22 (Valldeoriola et al., 2019; Xie et al., 2015), 24 (Anderson et al., 2005)], and 14 were published in 2020 or later [studies 1–5 (Seger et al., 2021; Bourilhon et al., 2022; Conway et al., 2021; Horn et al., 2021; Breit et al., 2023), 8–11 (Dutke et al., 2025; Cherif et al., 2024; Lizarraga et al., 2022; Kroneberg et al., 2024), 16 and 17 (Isaias et al., 2025; Artusi et al., 2024), 20 (Wilkins et al., 2025), 23 (Karl et al., 2020), 25 (Gharabaghi et al., 2024)], the number of studies and participants corresponding to each stimulation modality is presented in Supplementary Table S12. Across the included studies, 324 participants were assigned to the intervention groups and 324 to the control groups; specific characteristics are summarized in Supplementary Table S1, which also details the intervention strategies applied in each study and their corresponding outcome measures. The primary outcomes assessed in the included studies were gait speed, MDS-UPDRS Part III scores, and FOG-Q scores. The results of methodological quality assessment are presented in Figure 2. The quality assessment was conducted independently by two reviewers (YG and YH) after jointly reading the full text of each study. All methodological domains were rated using a three-tier scale (high, moderate, low). After completing the assessments, the ratings from all included studies were aggregated, and the proportions of high-, moderate-, and low-quality judgments were calculated to determine the overall quality grade. Based on this synthesis, the overall quality of the evidence was classified as moderate, which meets the methodological expectations for conducting a network meta-analysis. The detailed risk-of-bias plot is shown in Supplementary Figure S2.

### 3.2 MDS-UPDRS Part III scores

**Figure 3:**
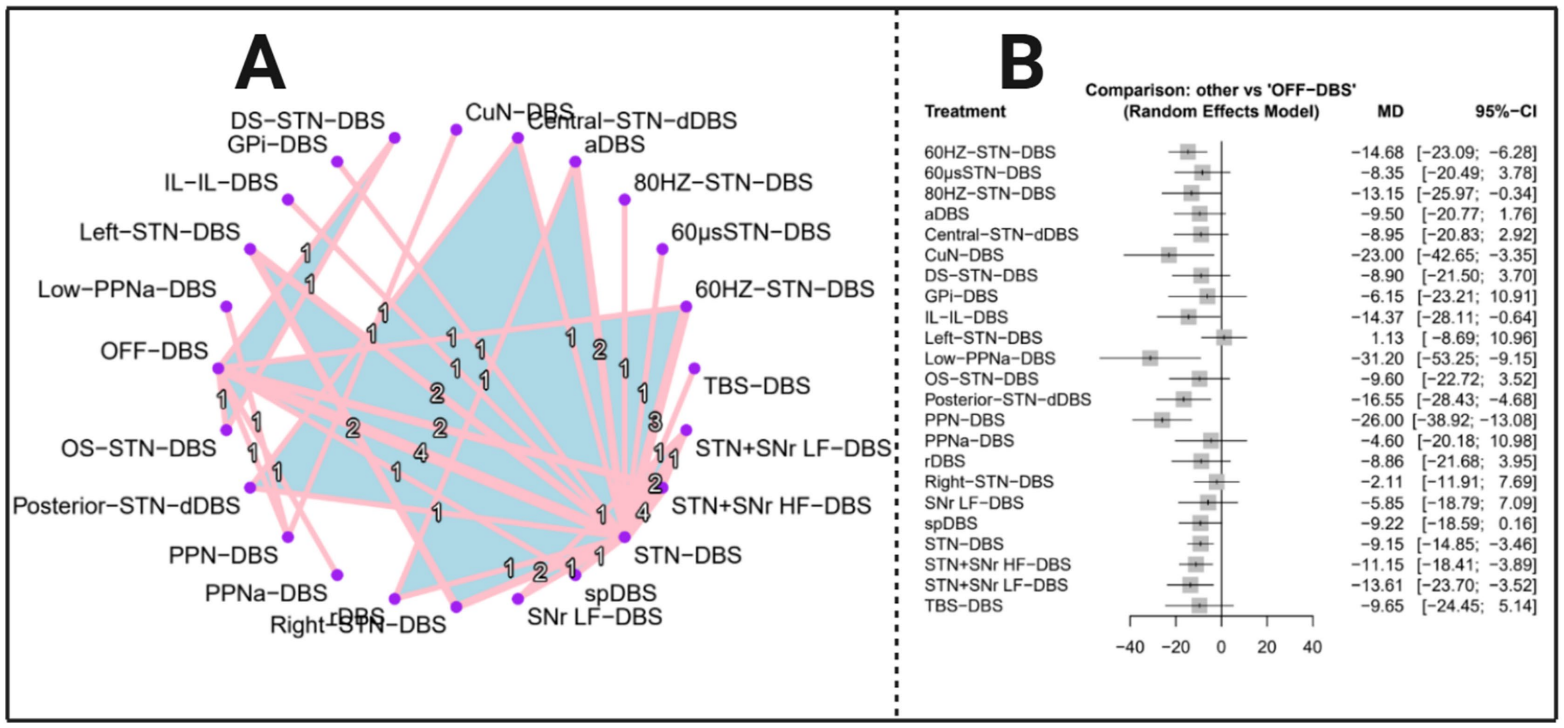
Network evidence diagram and total effect forest plot (outcome indicator: MDS-UPDRS III score). Panel A presents the network evidence plot of the network meta-analysis with MDS-UPDRS III as the outcome, while Panel B presents the corresponding forest plot.

The network evidence diagram (Figure 3A) demonstrated that 24 randomized controlled trials (1–6, 8–25) evaluated 23 types of deep brain stimulation (DBS), namely: 60 HZ-STN-DBS; 60 μs-STN-DBS; 80 HZ-STN-DBS; aDBS; Central-STN-dDBS; CuN-DBS; DS-STN-DBS; GPi-DBS; IL-IL-DBS; Left-STN-DBS; Low-PPNa-DBS; OS-STN-DBS; Posterior-STN-dDBS; PPN-DBS; PPNa-DBS; rDBS; Right-STN-DBS; SNr LF-DBS; spDBS; STN-DBS; STN + SNr HF-DBS; STN + SNr LF-DBS; TBS-DBS. The network forest plot (Figure 3B) presents the mean differences (MDs) and 95% confidence intervals (CIs) for all DBS interventions included in the NMA. Given that the global heterogeneity reached 68.7%, the random-effects model was designated as the primary analytical framework.

The results of the network meta-analysis (Table 2) present the mean differences (MDs), 95% confidence intervals (CIs), *z*-values, and *p*-values for all 23 DBS interventions on MDS-UPDRS Part III scores. Among the included treatments, Low-PPNa-DBS exhibited the most substantial therapeutic improvement [MD = −31.20, 95% CI (−53.25, −9.15), *p* = 0.0055], representing the largest statistically significant reduction in motor symptoms. PPN-DBS [MD = −26.00, 95% CI (−38.92, −13.08), *p* < 0.0001] and CuN-DBS [MD = −23.00, 95% CI (−42.65, −3.35), *p* = 0.0218] also demonstrated robust and clinically meaningful effects, with consistently negative MDs and narrow confidence intervals that do not cross zero.

**Table 2:** Comprehensive effect table of network meta-analysis (outcome indicators: MDS-UPDRS III score).

Treatment	MD	95% CI	*z*	*p*-value
60 HZ-STN-DBS	−14.6821	[−23.0852, −6.2789]	−3.42	0.0006***
60 μs-STN-DBS	−8.3543	[−20.4906, 3.7821]	−1.35	0.1773
80 HZ-STN-DBS	−13.1543	[−25.9675, −0.3410]	−2.01	0.0442*
aDBS	−9.5029	[−20.7695, 1.7637]	−1.65	0.0983
Central-STN-dDBS	−8.9543	[−20.8261, 2.9176]	−1.48	0.1393
CuN-DBS	−23	[−42.6506, −3.3494]	−2.29	0.0218*
DS-STN-DBS	−8.9	[−21.5012, 3.7012]	−1.38	0.1663
GPi-DBS	−6.1543	[−23.2146, 10.9061]	−0.71	0.4796
IL-IL-DBS	−14.3743	[−28.1085, −0.6400]	−2.05	0.0402*
Left-STN-DBS	1.1313	[−8.6925, 10.9552]	0.23	0.8214
Low-PPNa-DBS	−31.2	[−53.2476, −9.1524]	−2.77	0.0055**
OS-STN-DBS	−9.6	[−22.7212, 3.5212]	−1.43	0.1516
Posterior-STN-dDBS	−16.5543	[−28.4261, −4.6824]	−2.73	0.0063**
PPN-DBS	−26	[−38.9189, −13.0811]	−3.94	0.0001***
PPNa-DBS	−4.6	[−20.1809, 10.9809]	−0.58	0.5628
rDBS	−8.8633	[−21.6793, 3.9528]	−1.36	0.1753
Right-STN-DBS	−2.1121	[−11.9129, 7.6887]	−0.42	0.6727
SNr LF-DBS	−5.8507	[−18.7899, 7.0884]	−0.89	0.3755
spDBS	−9.2156	[−18.5904, 0.1592]	−1.93	0.054
STN-DBS	−9.1543	[−14.8491, −3.4594]	−3.15	0.0016**
STN + SNr HF-DBS	−11.1494	[−18.4103, −3.8884]	−3.01	0.0026**
STN + SNr LF-DBS	−13.6105	[−23.7001, −3.5209]	−2.64	0.0082**
TBS-DBS	−9.6543	[−24.4477, 5.1392]	−1.28	0.2009

① MD (mean difference): Difference in outcome between each DBS intervention and the reference group (OFF-DBS). ② 95% CI: 95% confidence interval. ③ *z*: Standardized test statistic. ④ *p*-value: *p* < 0.05 is considered statistically significant. ⑤ *: *p* < 0.05 indicates statistical significance; **: *p* < 0.01 indicates high significance; ***: *p* < 0.001 indicates very high significance.

Several other interventions showed significant but moderate improvements. Posterior-STN-dDBS [MD = −16.55, 95% CI (−28.43, −4.68), *p* = 0.0063] and IL-IL-DBS [MD = −14.37, 95% CI (−28.11, −0.64), *p* = 0.0402] yielded notable therapeutic benefits. 60 Hz-STN-DBS [MD = −14.68, 95% CI (−23.09, −6.28), *p* = 0.0006] and 80 Hz-STN-DBS [MD = −13.15, 95% CI (−25.97, −0.34), *p* = 0.0442] also showed statistically significant improvements, although the wider confidence intervals suggest potential variability across the contributing studies. Similarly, STN-DBS [MD = −9.15, 95% CI (−14.85, −3.46), *p* = 0.0016], STN + SNr HF-DBS [MD = −11.15, 95% CI (−18.41, −3.89), *p* = 0.0026], and STN + SNr LF-DBS [MD = −13.61, 95% CI (−23.70, −3.52), *p* = 0.0082] were associated with statistically significant reductions, representing consistent, moderate treatment effects.

By contrast, several interventions yielded non-significant results, primarily due to confidence intervals crossing zero or *p*-values exceeding 0.05. These included 60 μs-STN-DBS, aDBS, Central-STN-dDBS, DS-STN-DBS, GPi-DBS, OS-STN-DBS, PPNa-DBS, rDBS, TBS-DBS, SNr LF-DBS, Left-STN-DBS, and Right-STN-DBS, indicating limited or inconclusive evidence of therapeutic benefit. spDBS demonstrated a borderline effect (MD = −9.22, *p* = 0.054), suggesting a possible treatment advantage that did not reach statistical significance. Supplementary Tables S4, S5 lists the direct pairwise comparisons between each intervention and the control group and common-effects model results are in Supplementary Table S6.

The heterogeneity and inconsistency of the network were quantitatively assessed. The between-study variance was *τ*^2^ = 26.32 (*τ* = 5.13), with an *I*^2^ of 68.7% (95% CI: 44.4–82.4%), indicating a moderate degree of heterogeneity. The overall *Q* statistic was 38.33 (df = 12, *p* = 0.0001), suggesting significant variability across the network. Decomposition showed that within-design heterogeneity remained significant (*Q* = 15.74, df = 5, *p* = 0.0076), while between-design inconsistency was also detected (*Q* = 22.59, df = 7, *p* = 0.0020), reflecting discrepancies between direct and indirect evidence.

Design-specific analyzes revealed inconsistency within the Left-STN-DBS: Right-STN-DBS: STN-DBS (*Q* = 9.89, df = 2, *p* = 0.0071) and 60 Hz-STN-DBS: STN-DBS (*Q* = 5.69, df = 1, *p* = 0.0171) designs, whereas the OFF-DBS: STN-DBS: STN + SNr HF-DBS design demonstrated good internal consistency (*Q* = 0.16, df = 2, *p* = 0.9245). Detach analyzes further indicated that several designs—such as STN-DBS: STN + SNr HF-DBS: STN + SNr LF-DBS, 60 Hz-STN-DBS: STN-DBS, and aDBS: STN-DBS—substantially influenced the global inconsistency signal.

When re-evaluated under a full design-by-treatment interaction random-effects model, the between-design inconsistency was no longer significant (*Q* = 7.83, df = 7, *p* = 0.3475), suggesting that the observed inconsistency may be attributable to specific influential designs rather than systematic conflict within the network. The results of Egger’s regression test for publication bias yielded a z-value of −1.27 with a corresponding *p*-value of 0.2041, indicating no statistically significant evidence of funnel plot asymmetry. The estimated intercept at the limit as the standard error approaches zero was −5.49 (95% CI: −13.27 to 2.28), further suggesting that there is no strong indication of small-study effects or publication bias in the included studies.

### 3.3 FOG-Q score

**Figure 4:**
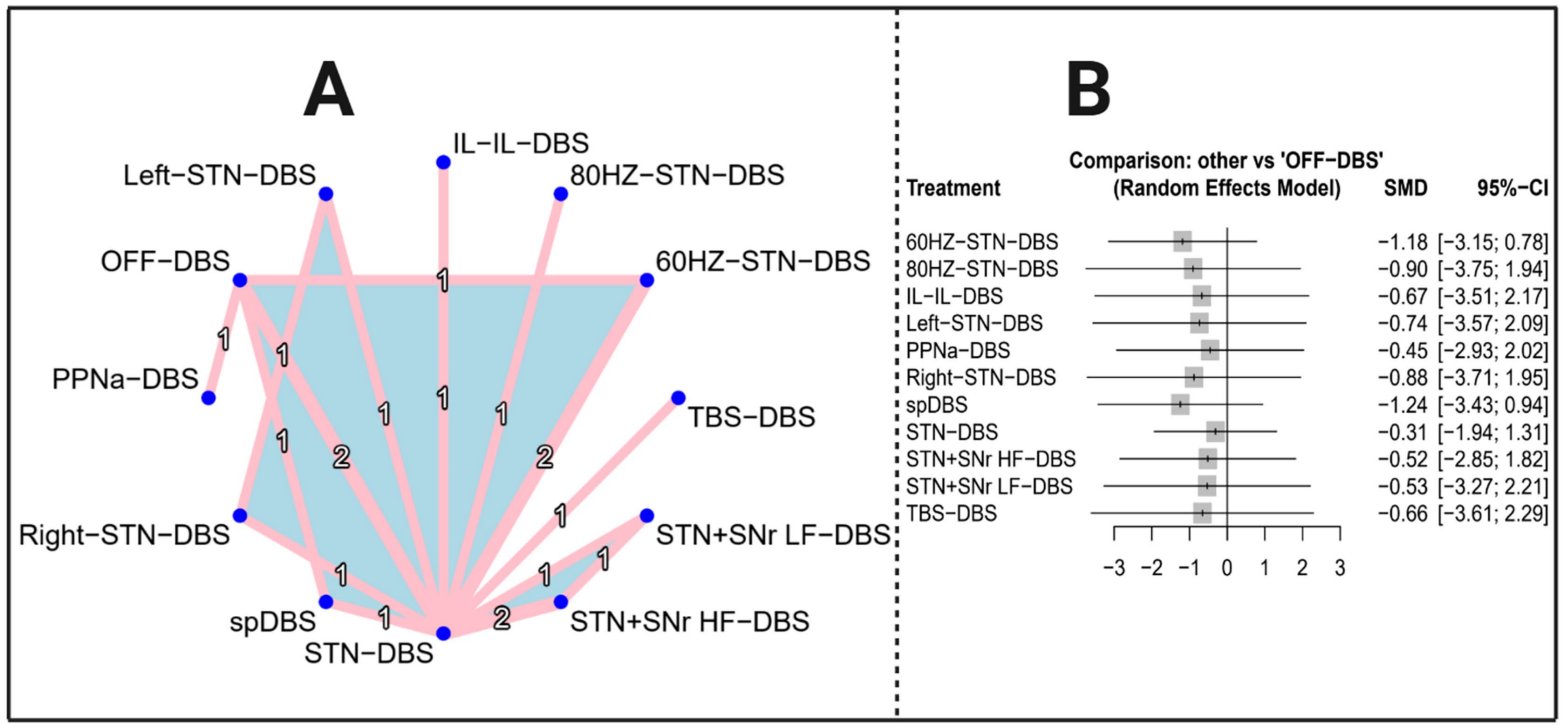
Network evidence diagram and forest plot (outcome indicator: FOG-Q score). Panel A presents the network evidence plot of the network meta-analysis with FOG-Q score as the outcome, while Panel B presents the corresponding forest plot.

The network evidence diagram (Figure 4A) depicts that the 10 included studies collectively evaluated a total of 11 distinct stimulation modalities: 60 Hz-STN-DBS, spDBS, 80 Hz-STN-DBS, right STN-DBS, left STN-DBS, IL-IL-DBS, TBS-DBS, STN + SNr LF-DBS, STN + SNr HF-DBS, PPNa-DBS, and STN-DBS. Table 3 presents the results of the network meta-analysis based on a random-effects model. The overall estimates indicate that, compared with the control group, none of the DBS interventions yielded statistically significant improvements in FOG-Q scores.

Table 3 presents the estimated effects of 11 DBS intervention modalities on FOG-Q scores included in this network meta-analysis (NMA), including mean differences (MD), 95% confidence intervals (CI), *z*-values, and *p*-values. The results indicate that the 95% CIs for all interventions cross zero, and none of the *p*-values reached statistical significance (*p* > 0.05), suggesting that no DBS intervention demonstrated a reliable statistical effect in improving FOG-Q scores; although all SMD values were negative and hinted at slight improvements, the overall strength of evidence remained limited. The corresponding visualized results are shown in Figure 4B. Consistency checks further demonstrated that differences between direct and indirect evidence were not statistically significant for any comparison (all *p* > 0.05), indicating good overall model consistency. Supplementary Tables S7, S8 lists the direct pairwise comparisons between each intervention and the control group.

**Table 3:** Comprehensive effect table of network meta-analysis (outcome: FOG-Q score).

Treatment	SMD	95% CI	*z*	*p*-value
60 HZ-STN-DBS	−1.1839	[−3.1479, 0.7801]	−1.18	0.2374
80 HZ-STN-DBS	−0.9037	[−3.7469, 1.9395]	−0.62	0.5333
IL-IL-DBS	−0.6721	[−3.5098, 2.1655]	−0.46	0.6425
Left-STN-DBS	−0.7368	[−3.5681, 2.0946]	−0.51	0.61
PPNa-DBS	−0.4548	[−2.9344, 2.0248]	−0.36	0.7193
Right-STN-DBS	−0.8819	[−3.7140, 1.9502]	−0.61	0.5416
spDBS	−1.2436	[−3.4306, 0.9434]	−1.11	0.2651
STN-DBS	−0.3117	[−1.9364, 1.3129]	−0.38	0.7068
STN + SNr HF-DBS	−0.5151	[−2.8478, 1.8175]	−0.43	0.6651
STN + SNr LF-DBS	−0.5314	[−3.2684, 2.2057]	−0.38	0.7036
TBS-DBS	−0.6592	[−3.6099, 2.2914]	−0.44	0.6615

Heterogeneity and consistency assessments for the FOG-Q outcome showed high variability, with *τ*^2^ = 1.3074 and *I*^2^ = 88.3% (95% CI: 72.5–95.0%). The total *Q* was 25.61 (df = 3, *p* < 0.0001), indicating significant network inconsistency. Within-design heterogeneity was negligible (*Q* = 0), while between-design heterogeneity was significant (*Q* = 25.61, df = 3, *p* < 0.0001), reflecting discrepancies between direct and indirect evidence. Detaching individual designs, OFF-DBS: 60 HZ-STN-DBS: STN-DBS showed no inconsistency (*Q* = 0.44, *p* = 0.5084), but 60 HZ-STN-DBS: STN-DBS (*Q* = 23.89, *p* < 0.0001) and STN-DBS: STN + SNr HF-DBS (*Q* = 25.17, *p* < 0.0001) remained inconsistent. The design-by-treatment interaction random-effects model confirmed that inconsistency mainly arose from differences between study designs.

Egger’s regression for the FOG-Q outcome indicated significant funnel plot asymmetry (*z* = 3.929, *p* < 0.0001), suggesting potential publication bias or small-study effects, with smaller studies tending to report higher scores (intercept = 5.971, 95% CI: 1.939–10.003), warranting cautious interpretation of the pooled estimates.

### 3.4 Gait speed (cm/s)

**Figure 5:**
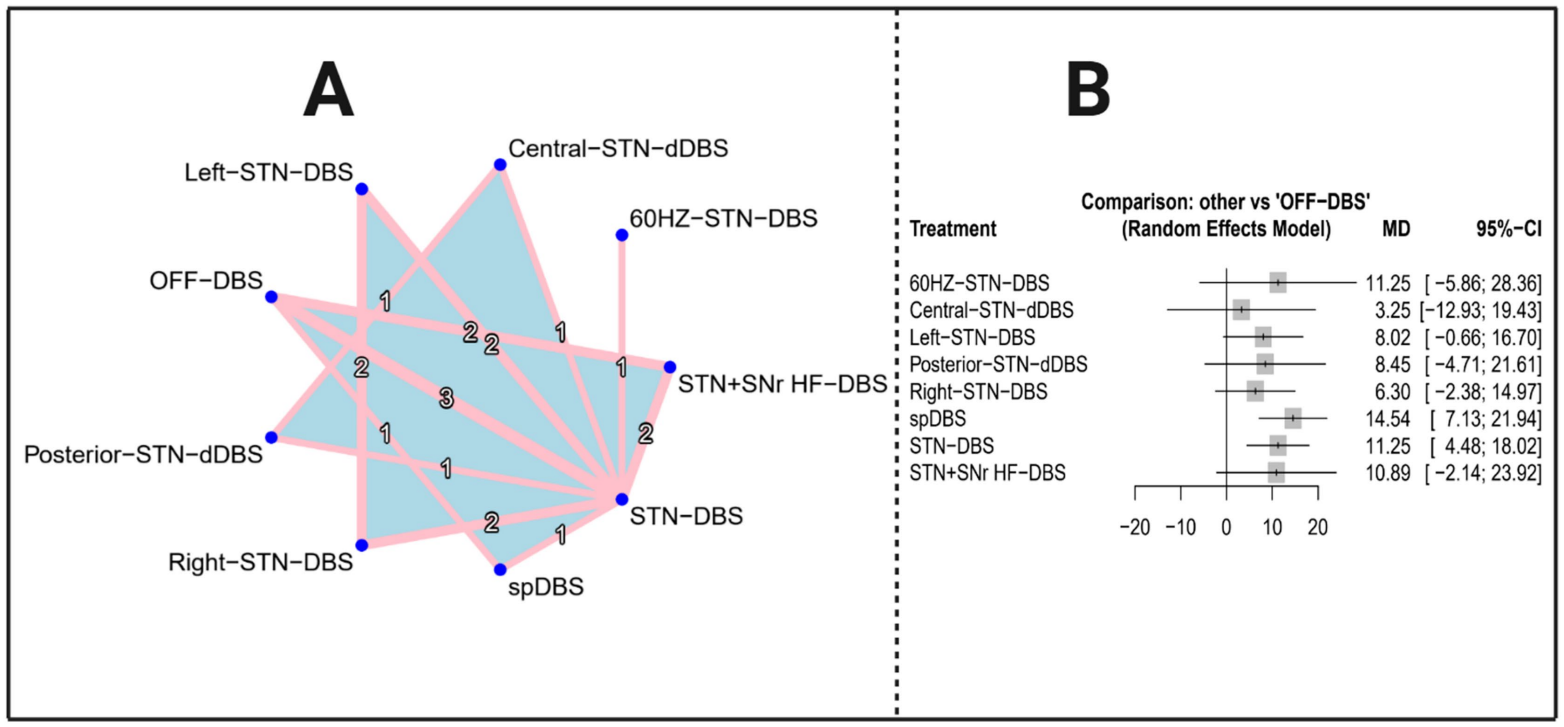
Network evidence plot and total effect forest plot (outcome indicator: gait speed). Panel A presents the network evidence plot of the network meta-analysis with Gait speed as the outcome, while Panel B presents the corresponding forest plot.

The network evidence diagram (Figure 5A) illustrates eight DBS modalities examined in 7 studies (1, 3, 4, 9–11, 18), including 60 HZ-STN-DBS; Central-STN-dDBS; Left-STN-DBS; Posterior-STN-dDBS; Right-STN-DBS; spDBS; STN-DBS; STN + SNr HF-DBS. The network forest plot (Figure 5B) displays the MDs and 95% CIs for all DBS interventions included in this NMA.

The results of the network meta-analysis present the mean differences (MDs), 95% confidence intervals (CIs), *z*-values, and *p*-values for all 8 DBS interventions on Gait Speed compared to OFF-DBS results are shown in Table 4. Among the included treatments, spDBS exhibited the most substantial therapeutic improvement [MD = 14.5369, 95% CI (7.1313; 21.9425), *p* = 0.0001], representing the largest statistically significant reduction in motor symptoms. STN-DBS [MD = 11.2475, 95% CI (4.4782; 18.0168), *p* = 0.0011] also demonstrated robust and clinically meaningful effects, with consistently positive MDs and narrow confidence intervals that do not cross zero.

By contrast, several interventions yielded non-significant results, primarily due to confidence intervals crossing zero or *p*-values exceeding 0.05. These included 60 HZ-STN-DBS [MD = 11.2475, 95% CI (−5.8632; 28.3582), *p* = 0.1976], Central-STN-dDBS [MD = 3.2475, 95% CI (−12.9307; 19.4256), *p* = 0.6940], Posterior-STN-dDBS [MD = 8.4475, 95% CI (−4.7140; 21.6090), *p* = 0.2084], Right-STN-DBS [MD = 6.2951, 95% CI (−2.3834; 14.9737), *p* = 0.1551], and STN + SNr HF-DBS [MD = 10.8890, 95% CI (−2.1404; 23.9184), *p* = 0.1014], indicating limited or inconclusive evidence of therapeutic benefit. Left-STN-DBS demonstrated a borderline effect [MD = 8.0182, 95% CI (−0.6604; 16.6968), *p* = 0.0702], suggesting a possible treatment advantage that did not reach statistical significance (see Table 4).

**Table 4:** Comprehensive effect table of network meta-analysis (outcome: gait speed).

Treatment	MD	95% CI	*z*	p-value
60 HZ-STN-DBS	11.2475	[−5.8632, 28.3582]	1.29	0.1976
Central-STN-dDBS	3.2475	[−12.9307, 19.4256]	0.39	0.694
Left-STN-DBS	8.0182	[−0.6604, 16.6968]	1.81	0.0702
Posterior-STN-dDBS	8.4475	[−4.7140, 21.6090]	1.26	0.2084
Right-STN-DBS	6.2951	[−2.3834, 14.9737]	1.42	0.1551
spDBS	14.5369	[7.1313, 21.9425]	3.85	0.0001***
STN-DBS	11.2475	[4.4782, 18.0168]	3.26	0.0011**
STN + SNr HF-DBS	10.889	[−2.1404, 23.9184]	1.64	0.1014

*: *p* < 0.05 indicates statistical significance; **: *p* < 0.01 indicates high significance; ***: *p* < 0.001 indicates very high significance.

The network meta-analysis indicated a moderate to substantial level of heterogeneity. The heterogeneity estimates were *τ*^2^ = 12.1151 and *τ* = 3.4807, with an *I*^2^ value of 65.5% (95% CI: 17.2–85.6%), suggesting that a considerable proportion of the observed variability was attributable to differences across studies. The overall test for heterogeneity yielded a significant result (*Q* = 14.48, df = 5, *p* = 0.0128). Examination of within-design heterogeneity also revealed significant variability (*Q* = 12.83, df = 4, *p* = 0.0121). In contrast, the test for between-design inconsistency was not statistically significant (*Q* = 1.65, df = 1, *p* = 0.1994), indicating no evidence of inconsistency between different study designs. Supplementary Tables S9, S10 lists the direct pairwise comparisons between each intervention and the control group and common-effects model results (Supplementary Table S11).

The regression test for funnel plot asymmetry, using standard error as the predictor in a mixed-effects meta-regression model, indicated no evidence of publication bias (*z* = −0.9069, *p* = 0.3645). The estimated effect as the standard error approached zero was 95.21 (95% CI: 78.19, 112.23).

Design-specific decomposition indicated that heterogeneity was primarily contributed by the Left-STN-DBS: Right-STN-DBS: STN-DBS comparison (*Q* = 12.55, df = 2, *p* = 0.0019), whereas the OFF-DBS: STN-DBS: STN + SNr HF-DBS design showed no significant heterogeneity (*Q* = 0.29, df = 2, *p* = 0.8660). Consistency across designs under a full design-by-treatment interaction random effects model was supported (*Q* = 1.14, df = 1, *p* = 0.2846), with a within-design Tau of 3.44 and Tau^2^ of 11.81, indicating moderate within-design random-effects variability.

### 3.5 P-score ranking

**Figure 6:**
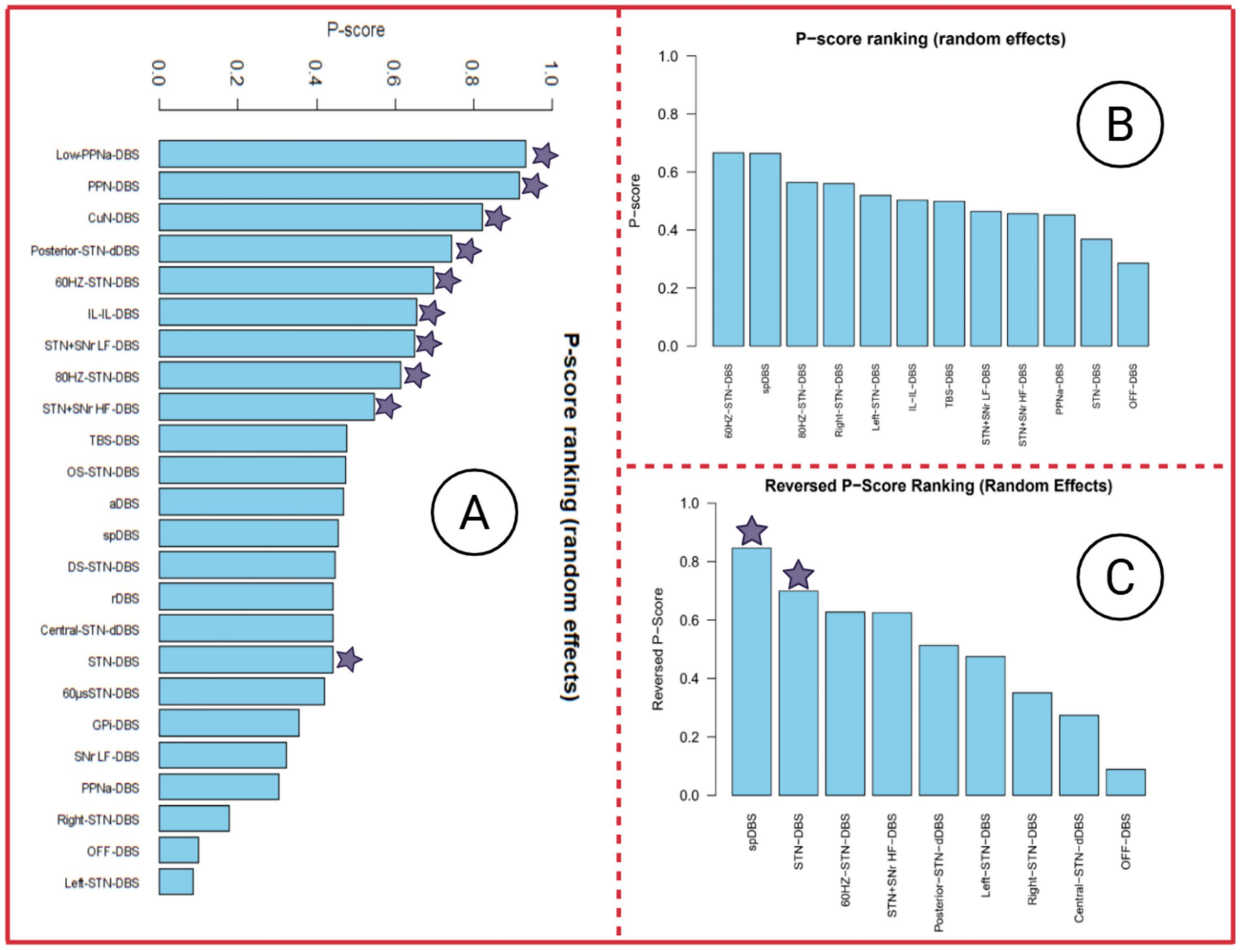
P-score ranking of three outcome indicators. In the P-score ranking table, DBS stimulation modes marked with a purple star indicate a significant improvement in this outcome measure, with *p* < 0.05. Panel A presents the P-score ranking with MDS-UPDRS III as the outcome, Panel B presents the P-score ranking with FOG-Q score as the outcome, and Panel C presents the P-score ranking with Gait speed as the outcome.

Figures 6A–C present the P-score rankings based on MDS-UPDRS III, FOG-Q, and gait speed as outcome measures, respectively. Notably, although none of the DBS stimulation modes reached statistical significance for the FOG-Q score outcome, the ranking suggests that certain DBS interventions may still confer potential improvements in FOG-Q scores. Therefore, these rankings may provide useful, albeit exploratory, reference for interpreting the relative efficacy of different stimulation strategies.

### 3.6 Sensitivity analysis, meta-regression, and subgroup analysis

#### 3.6.1 Sensitivity analysis

**Figure 7:**
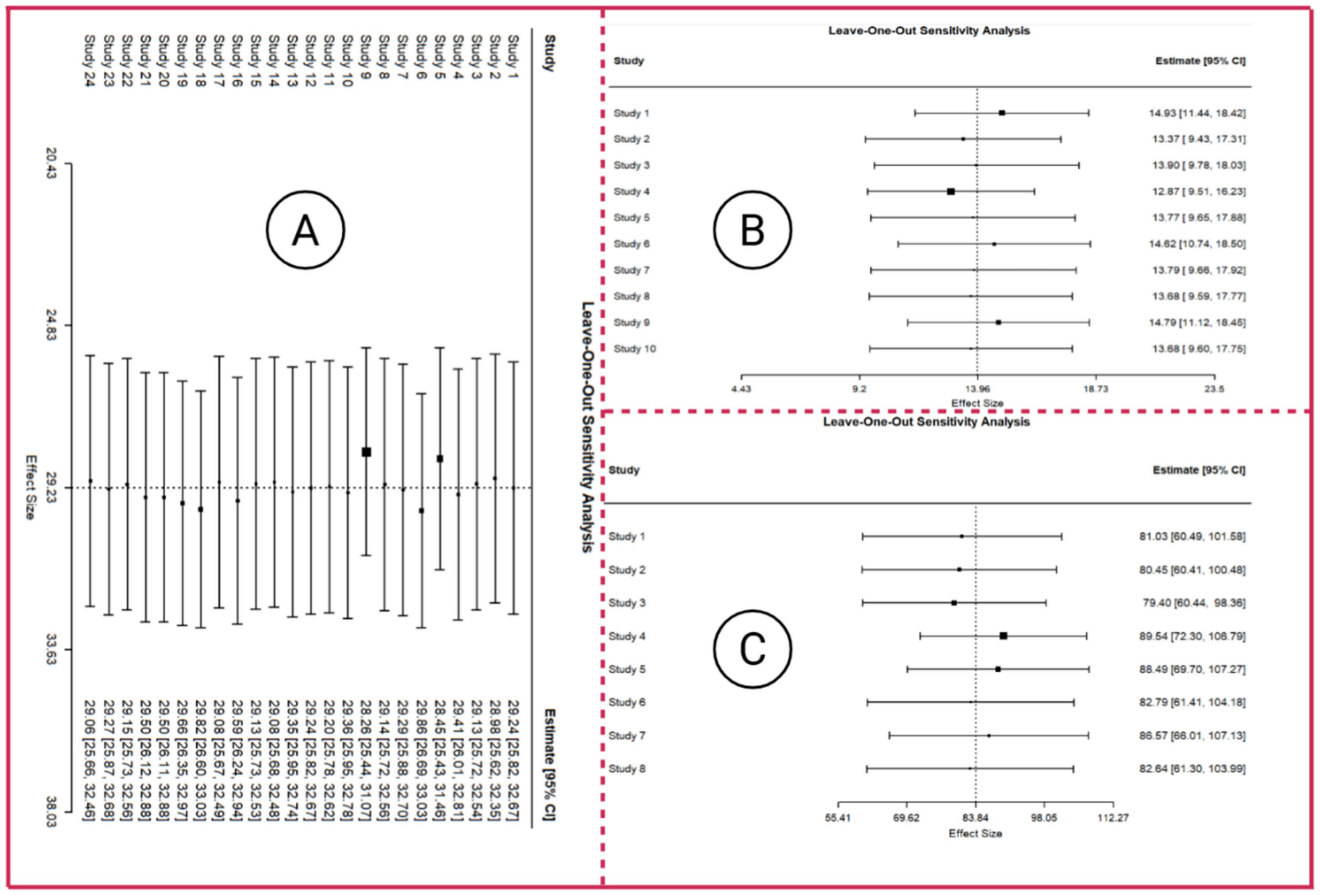
Forest plot of leave-one-out sensitivity analysis. Panel A presents the leave-one-out sensitivity analysis with MDS-UPDRS III as the outcome, Panel B presents the leave-one-out sensitivity analysis with FOG-Q score as the outcome, and Panel C presents the leave-one-out sensitivity analysis with Gait speed as the outcome.

① For MDS-UPDRS III score, the leave-one-out sensitivity analyses produced pooled estimates ranging from 28.26 to 29.86, showing only minimal variation after sequentially omitting individual studies and thereby supporting the stability of the overall effect. All 95% confidence intervals consistently remained above zero, and every *p*-value was <0.001, indicating that the intervention effect retained clear statistical significance throughout the analyses. Correspondingly, *z*-values varied between 16.70 and 19.68, further confirming the robustness and precision of the estimated effects. Substantial heterogeneity persisted across most iterations. Heterogeneity levels showed minimal fluctuation across all iterations, indicating that sequentially removing individual studies did not materially influence between-study variability. The associated forest plot for the sensitivity analysis is presented in Figure 7A.
② For the FOG-Q outcome, the leave-one-out sensitivity analysis demonstrated that omitting any single study resulted in minimal changes in the pooled FOG-Q estimates (range approximately 12.9–14.9), with 95% confidence intervals remaining non-crossing zero, indicating robust findings, and all *p*-values were <0.05, confirming statistical significance of the intervention. High heterogeneity metrics (*I*^2^ > 90%, significant Q statistics, and substantial Tau^2^) suggest extensive between-study variability; however, these differences are not driven by any individual study and have limited impact on the overall results. The leave-one-out forest plot is displayed in Figure 7B.
③ For gait speed, leave-one-out sensitivity analysis results showed that the overall effect estimates ranged from 79.40 to 89.54, exhibiting only modest variation after sequentially omitting individual studies and thereby supporting the stability of the pooled effect. All 95% confidence intervals remained clearly above zero, and all *p*-values were <0.0001, confirming that the intervention effect consistently retained statistical significance. Although heterogeneity remained extremely high across all iterations, shifts in the *Q*-statistics and *I*^2^ values were minimal, indicating that no single study exerted a disproportionate impact on between-study variability. The corresponding leave-one-out forest plot (Figure 7C) further illustrates the limited fluctuation in effect sizes.supporting the reliability and robustness of the network meta-analysis results.

#### 3.6.2 Meta-regression

Subgroup analyses and Meta-regression analysis were conducted for MDS-UPDRS III (23 studies: 1–6, 8–17, 19–25), FOG-Q (studies 1, 2, 5, 6, 8, 10–12, 17, 22, 23), and gait speed (studies 1, 3, 4, 7, 9–11). Moderators included in the analyses were age, disease duration, male prevalence, and the reporting of a washout period. Male ratio and disease duration were dichotomized by their medians to create balanced subgroups, avoiding arbitrary cut-offs and enabling exploratory assessment of potential effect modification (Rubio-Aparicio et al., 2017). The cutoff of 60 years was selected based on common clinical and epidemiological conventions in Parkinson’s disease and neurological research, distinguishing middle-aged individuals from older populations. This threshold allows for meaningful subgroup comparisons while maintaining sufficient study numbers per group (Pringsheim et al., 2014; Macleod et al., 2018). The median disease duration was 13.7 years, and the median male ratio was 77.8%. Funnel plots for the three outcome measures are presented in Supplementary Figure S3, and the meta-regression results are shown in Supplementary Figure S4.

① For MDS-UPDRS III score (results are shown in Supplementary Figure S4A). The meta-regression, comprising 56 effect sizes, indicated substantial residual heterogeneity (*τ*^2^ = 23.45; *I*^2^ = 72.69%), with none of the included study-level moderators accounting for variability (*R*^2^ = 0%). Individual predictors—including washout status, male proportion, disease duration, and age—demonstrated no statistically meaningful impact on the outcome (all *p* > 0.16). The intercept was also non-significant (estimate = −4.20; *p* = 0.115), suggesting a lack of consistent baseline effect across studies. These results imply that the heterogeneity observed across studies is largely unexplained by the examined covariates, highlighting the potential influence of other unmeasured or study-specific factors on the outcome.
② For FOG-Q score (results are shown in Supplementary Figure S4B). The meta-regression found moderate residual heterogeneity (*τ*^2^ = 1.96; *I*^2^ = 46.27%) with only 20.4% explained by washout status, male proportion, disease duration, and age. None of these moderators reached statistical significance (all *p* > 0.22), and the intercept was not significant (estimate = −4.53; *p* = 0.143), indicating that these study-level factors did not meaningfully influence the treatment effect. The persistent heterogeneity suggests that other unmeasured variables may underlie between-study differences.
③ For gait speed (results are shown in Supplementary Figure S4C). Substantial residual heterogeneity remained (*τ*^2^ = 27.16, *I*^2^ = 82.0%), with the included moderators explaining 30.0% of between-study variability. Among the factors examined, shorter disease duration was associated with greater gait improvement (estimate = 10.29; *p* = 0.0048), whereas washout period, male proportion, and age showed no significant influence. These results indicate that disease duration is a key moderator of DBS efficacy on gait speed, while the other factors contribute little to variability, and persistent high heterogeneity suggests additional unmeasured study-level factors may affect treatment response.

#### 3.6.3 Subgroup analysis

① A series of subgroup analyses was conducted to explore potential moderators of the treatment effect on MDS-UPDRS III scores, exhibition of the results of subgroup analysis in Table 5. Overall, none of the subgroup stratifications substantially reduced heterogeneity, and Tau^2^ and *I*^2^ remained consistently high across all models (Tau^2^ ≈ 31–36; *I*^2^ ≈ 83–86%), indicating persistent and substantial between-study variability. Across all subgroup analyses, male ratio and disease duration showed the clearest moderating patterns, with statistically significant effects detected in the high-male and short-duration strata. Age demonstrated a borderline trend, while washout period exhibited no moderating influence. Persistent high heterogeneity across all models suggests that these moderators explain only a limited proportion of between-study variability, and results should therefore be interpreted with caution.
② A series of subgroup analyses was conducted to explore potential moderators of the treatment effect on FOG-Q scores, with results summarized in Table 6. Across all subgroup analyses, male ratio and washout period demonstrated significant moderating patterns: the low male and washout no subgroups showed statistically significant reductions in FOG-Q scores (estimate = −1.58, *p* = 0.005; estimate = −1.40, *p* = 0.018, respectively), whereas other subgroups did not reach significance. Disease duration and age showed only borderline or non-significant trends, with the short disease subgroup trending toward greater improvement (estimate = −1.18, *p* = 0.081) and younger participants showing a non-significant tendency for benefit (estimate = −2.90, *p* = 0.200).
③ Subgroup analyses were performed to explore whether washout period, male proportion, disease duration, or age could moderate the effect of treatment on FOG-Q scores, with results presented in Table 7. No subgroup reached statistical significance (all *p* > 0.05), indicating that these factors did not substantially influence the overall treatment effect. Effect estimates varied between −0.65 and 4.10, with wide and overlapping confidence intervals, reflecting considerable uncertainty in the subgroup-specific outcomes. Residual heterogeneity remained high across all analyses (Tau^2^ ≈ 34–39; *I*^2^ ≈ 83–86%), suggesting that these variables account for only a small fraction of the between-study variability.

**Table 5:** Subgroup analysis of MDS-UPDRS III score.

Indicator	Subgroup	*k*	Estimate	SE	*z*-value	*p*	95% CI (lb, ub)	Tau^2^	*I*^2^ (%)	*H* ^2^
Report washout period	N/A	59	−0.8425	4.2131	−0.2	0.8415	−9.1000, 7.4150	36.1449	85.83	7.06
	No	59	−2.0541	1.2009	−1.71	0.0872	−4.4078, 0.2997	36.1449	85.83	7.06
	Yes	59	−2.7864	1.5595	−1.787	0.074	−5.8429, 0.2701	36.1449	85.83	7.06
Male ratio	High male %	59	−3.3938	1.4254	−2.381	0.0173*	−6.1876, −0.6001	34.5894	85.14	6.73
	Low male %	59	−1.4493	1.1892	−1.219	0.2229	−3.7800, 0.8814	34.5894	85.14	6.73
Disease duration	Long	59	0.1124	1.349	0.083	0.9336	−2.5315, 2.7564	31.0554	83.39	6.02
	Short	59	−3.9477	1.1563	−3.414	0.0006***	−6.2141, −1.6814	31.0554	83.39	6.02
Mean age	Old	59	−1.833	1.0306	−1.779	0.0753	−3.8529, 0.1868	34.9122	85.66	6.97
	Young	59	−3.8179	2.0017	−1.907	0.0565	−7.7412, 0.1055	34.9122	85.66	6.97

**Table 6:** Subgroup analysis of FOG-Q score.

Indicator	Subgroup	*k*	Estimate	SE	*z*-value	*p*	95% CI (lb, ub)	Tau^2^	*I*^2^ (%)	*H* ^2^
Report washout period	N/A	26	−1.7633	3.8391	−0.4593	0.646	−9.2877, 5.7612	2.2691	47.59	1.91
	No	26	−1.3999	0.5918	−2.3654	0.018*	−2.5599, −0.2399	2.2691	47.59	1.91
	Yes	26	0.2213	1.1021	0.2008	0.8408	−1.9387, 2.3814	2.2691	47.59	1.91
Male ratio	High male	26	0.291	0.9361	0.3109	0.7559	−1.5436, 2.1256	1.6695	39.18	1.64
	Low male	26	−1.5826	0.56	−2.826	0.0047**	−2.6803, −0.4850	1.6695	39.18	1.64
Disease duration	Long	26	−0.8387	0.8272	−1.0138	0.3107	−2.4600, 0.7826	2.4015	47.45	1.9
	Short	26	−1.182	0.677	−1.7459	0.0808	−2.5090, 0.1449	2.4015	47.45	1.9
Mean age	Old	26	−0.9444	0.5341	0.0770	0.077	−1.9912, 0.1023	2.3254	48.27	1.93
	Young	26	−2.8986	2.2625	0.2001	0.2001	−7.3329, 1.5358	2.3254	48.27	1.93

**Table 7:** Subgroup analysis of gait speed.

Indicator	Subgroup	*k*	Estimate	SE	*z*-value	*p*	95% CI (lb, ub)	Tau^2^	*I*^2^ (%)	*H* ^2^
Report washout period	No	22	2.2661	1.8786	1.2062	0.2277	(−1.4160, 5.9481)	39.3422	86.2	7.25
	Yes	22	−0.3816	3.3905	−0.1126	0.9104	(−7.0270, 6.2637)	39.3422	86.2	7.25
Male	High male	22	−0.0941	3.0701	−0.0307	0.9755	(−6.1114, 5.9231)	39.4706	86.24	7.27
	Low male	22	2.3443	1.9488	1.2029	0.229	(−1.4753, 6.1638)	39.4706	86.24	7.27
Male ratio	Long disease	22	−0.6517	2.1695	−0.3004	0.7639	(−4.9039, 3.6006)	34.1087	83.54	6.07
	Short disease	22	4.0961	2.232	1.8351	0.0665	(−0.2786, 8.4708)	34.1087	83.54	6.07
Mean age	Old	22	2.0829	1.8017	1.1561	0.2477	(−1.4484, 5.6142)	39.3744	86.22	7.26
	Young	22	−0.5344	4.0145	−0.1331	0.8941	(−8.4027, 7.3339)	39.3744	86.22	7.26

The notation in Tables 5–7 is as follows: *: *p* < 0.05 indicates statistical significance; **: *p* < 0.01 indicates high significance; ***: *p* < 0.001 indicates very high significance.

## 4 Discussion

### 4.1 Principal findings and clinical implications

This systematic review and network meta-analysis (NMA) provides a comprehensive evaluation of the comparative efficacy of various deep brain stimulation (DBS) modalities on motor and gait function in Parkinson’s disease (PD). Following the PICOS framework and PRISMA guidelines, 25 studies were included. Future research may consider revising certain exclusion criteria with minimal impact on study validity to enhance the comprehensiveness of evidence synthesis. Our findings indicate differential efficacy across DBS targets and parameters: Low-PPNa-DBS, PPN-DBS, and CuN-DBS emerged as potentially beneficial for general motor impairment (MDS-UPDRS Part III), though evidence remains limited due to small sample sizes. This further supports that, in patients with Parkinson’s disease (PD), postoperative quality of life and motor and non-motor symptoms largely depend on DBS electrode implantation location and stimulation parameters (Moro et al., 2002; Horn et al., 2017; Petry-Schmelzer et al., 2019; Anderson et al., 2020; Anderson et al., 2021). Short-pulse DBS (spDBS) showed the most promising effects for improving gait speed among included studies, followed by conventional STN-DBS, this finding provides supporting evidence that shorter pulse widths broaden the therapeutic window of subthalamic nucleus stimulation (Reich et al., 2015; Bouthour et al., 2018; Dayal et al., 2020).

Notably, none of the DBS modalities yielded statistically significant improvements in Freezing of Gait (FOG) as measured by the FOG-Q, with all confidence intervals crossing zero. This finding reflects the multifactorial and episodic nature of FOG (Ehgoetz Martens et al., 2018; Weiss et al., 2020), which is likely mediated by extranigral circuits such as the pedunculopontine nucleus and mesencephalic locomotor region (Wojcik et al., 2025; Factor et al., 2025; Camastra et al., 2025), a meta-analyses (Schlenstedt et al., 2017) suggest that STN-DBS improves gait and FOG in the medication-off/stimulation-on state, suggesting that the observed lack of significant effect in this study may be influenced by variations in study design, stimulation parameters, or patient characteristics. The dissociation between improvements in continuous gait parameters and persistent FOG episodes emphasizes the need to differentiate between continuous motor control and episodic gait disturbances in DBS evaluations. Future studies should consider tailored stimulation strategies, including alternative targets or parameter modulation, to specifically address FOG, because evidence indicates that low-frequency stimulation (LFS, ~60 Hz) may improve freezing of gait (FOG) in Parkinson’s disease patients treated with bilateral STN-DBS (Xie et al., 2017; Blumenfeld et al., 2017), thereby enhancing the translational relevance of DBS for complex gait impairments in PD.

### 4.2 Robustness of evidence and heterogeneity

Methodological rigor was a cornerstone of this investigation. Despite this statistical stability, we observed moderate-to-high heterogeneity. Our subgroup and meta-regression analyses identified disease duration as a significant moderator, with shorter disease duration associated with greater improvements in gait speed. This suggests a “window of opportunity” for DBS intervention, where earlier treatment—before the development of refractory axial symptoms or extensive non-dopaminergic degeneration—may yield superior functional mobility outcomes. Additionally, the apparent publication bias observed in FOG-Q outcomes (Egger’s test *p* < 0.0001) should be interpreted cautiously, given the limited number of studies per outcome, implying that smaller negative studies may remain unpublished and potentially inflate the perceived efficacy in the broader literature.

### 4.3 Limitations

While this NMA offers high-level evidence, several limitations must be acknowledged. First, owing to missing data in some reports, we were unable to include some key parameters—preoperative motor severity and electrode placement etc.—in subgroup or regression analyses. Second, non-motor outcomes, such as cognition and mood (Poewe, 2008), were not systematically analyzed due to inconsistent or incomplete reporting in the included studies. These outcomes are clinically relevant and may be differentially affected by DBS modalities. Future research should more consistently assess and report non-motor outcomes to enable a comprehensive evaluation of DBS effects. Thirdly, because gait variability was not assessed in the majority of the included studies, this important outcome could not be analyzed in the present review; future systematic reviews should place greater emphasis on evaluating the effects of DBS on gait variability.

### 4.4 Future directions

The field of neuromodulation for PD gait disorders stands at a critical juncture. The dissociation between motor score improvements and persistent freezing episodes necessitates a paradigm shift in target selection and stimulation paradigms. Future research should focus on the mechanistic effects of DBS at different targets on PD gait disturbances, clarifying the roles of specific targets in neural circuit modulation, motor function improvement, and gait parameter changes. Specifically, combined stimulation strategies warrant further investigation, though current evidence is preliminary and limited. Moreover, current studies display methodological inconsistencies in gait-related outcome assessment. The adoption of objective, wearable sensor-based gait analysis, rather than reliance solely on subjective scales like the FOG-Q, represents a vital step toward precision medicine in functional neurosurgery.

## 5 Conclusion

Across multiple network meta-analyses, distinct DBS modalities demonstrated differential efficacy across motor and gait-related outcomes. For MDS-UPDRS Part III, Low-PPNa-DBS, PPN-DBS, and CuN-DBS yielded the most pronounced and statistically robust improvements, whereas several other STN-targeted interventions achieved moderate but meaningful reductions in motor symptoms. In contrast, analysis of FOG-Q scores revealed no statistically significant effects for any DBS intervention, with all confidence intervals crossing zero, indicating limited and inconclusive benefits for freezing of gait. Regarding gait speed, spDBS and STN-DBS showed clear and significant enhancements, while other modalities produced non-significant or borderline effects, suggesting variability in efficacy across interventions. Collectively, these findings underscore that while certain DBS approaches confer substantial motor and gait improvements, their impact on FOG remains uncertain, and heterogeneity in response highlights the need for individualized treatment considerations and further high-quality investigations.

## Data Availability

The original contributions presented in the study are included in the article/Supplementary material, further inquiries can be directed to the corresponding author.

## Footnotes

1
https://automeris.io/WebPlotDigitizer

## Glossary

60 Hz-STN-DBS: Low-frequency (60 Hz) subthalamic nucleus deep brain stimulation
60 μs-STN-DBS: 60-Microsecond short-pulse subthalamic nucleus deep brain stimulation
80 Hz-STN-DBS: Low-frequency (80 Hz) subthalamic nucleus deep brain stimulation
aDBS: Adaptive deep brain stimulation
Central-STN-dDBS: Central subthalamic nucleus directional deep brain stimulation
CuN-DBS: Cuneiform nucleus deep brain stimulation
DB-Crossover RCT: Randomized crossover trial, double-blind
DB-RCT: Randomized controlled trial, double-blind
DBS: Deep brain stimulation
DS-STN-DBS: Directional subthalamic nucleus deep brain stimulation
GPi-DBS: Globus pallidus internus deep brain stimulation
IL-IL-DBS: Interleave-interlink, a dual-frequency DBS programming paradigm
Left-STN-DBS/Right-STN-DBS: Unilateral subthalamic nucleus deep brain stimulation (left or right hemisphere)
Low-PPNa-DBS: Low-frequency bilateral pedunculopontine nucleus area deep brain stimulation
OFF-DBS: Deep brain stimulation turned off (control condition)
OS-STN-DBS: Omnidirectional subthalamic nucleus deep brain stimulation
PPNa-DBS: Bilateral pedunculopontine nucleus area deep brain stimulation
PPN-DBS: Pedunculopontine nucleus deep brain stimulation
Posterior-STN-dDBS: Posterior subthalamic nucleus directional deep brain stimulation
rDBS: Randomly adapting deep brain stimulation
Sham-DBS: Sham deep brain stimulation (placebo condition)
SNr LF-DBS: Low-frequency substantia nigra reticulata deep brain stimulation
STN-DBS: Subthalamic nucleus deep brain stimulation
STN + SNr HF-DBS: High-frequency combined subthalamic nucleus and substantia nigra reticulata deep brain stimulation
STN + SNr LF-DBS: Low-frequency combined subthalamic nucleus and substantia nigra reticulata deep brain stimulation
STN + SNr-DBS: Combined subthalamic nucleus and substantia nigra reticulata deep brain stimulation
spDBS: Short-pulse (40 μs) subthalamic nucleus deep brain stimulation
TBS-DBS: Theta burst stimulation combined with deep brain stimulation
UPDRS III: Unified Parkinson’s Disease Rating Scale, Part III (motor examination)
FOG-Q: Freezing of Gait Questionnaire
