# Supplementary material for: Effects of different types of deep brain stimulation on gait disorders in patients with Parkinson’s disease: a network meta-analysis of randomized controlled trials
Source: Front Aging Neurosci. 2026 Jan 22;17:1723706. doi: 10.3389/fnagi.2025.1723706 (PMC12872896; doi:10.3389/fnagi.2025.1723706)
Supplement: Supplementary file 1 [file Data_Sheet_1.docx]

**Supplement**

***Table S1: Basic Information of All Included Literature***

***Table S2: Specific outcome data reported in the included studies***

***Table S3: Search strategies for each database***

***Table S4: Pairwise Comparisons under the Common-Effects Model (Outcome Indicator: ①)***

***Table S5: Pairwise Comparisons under the Random-Effects Model (Outcome Indicator: ①)***

***Table S6:*** ***Common-Effects Model: Treatment Effects vs OFF-DBS (Outcome Indicator: ①)***

***Table S7: Pairwise Comparisons under the Random-Effects Model (Outcome Indicator: ②)***

***Table S8：Pairwise Comparisons under the Random-Effects Model (Outcome Indicator: ②)***

***Table S9：Pairwise Comparisons under the Common-Effects Model (Outcome Indicator: ③)***

***Table S10：Pairwise Comparisons under the Random-Effects Model (Outcome Indicator: ③)***

***Table S11：Common-Effects Model: Treatment Effects vs OFF-DBS (Outcome Indicator: ③)***

***Table S12：The number of studies and participants corresponding to each stimulation***

***Supplementary Figure S 1：Search results of various databases***

***Supplementary Figure S 2：Risk assessment diagram of 25 articles.***

***Supplementary Figure S 3：Bias funnel plot：outcome indicators are A:①/ B:②/ C:③***

***Supplementary Figure S 4：Meta-regression analysis results: Outcome A:①/B:②/C:③***

***Note S1：Rationale for Excluding CINAHL from the Final Search Strategy
Note S2: Inclusion of a Newly Published Study Beyond the Protocol Cutoff Date
Note S3: Computation of Standardized Mean Differences (SMD/Hedges’ g)***

***In the appendix, outcome measures ①, ②, and ③ refer to UPDRS-III, FOG-Q, and gait speed, respectively.***


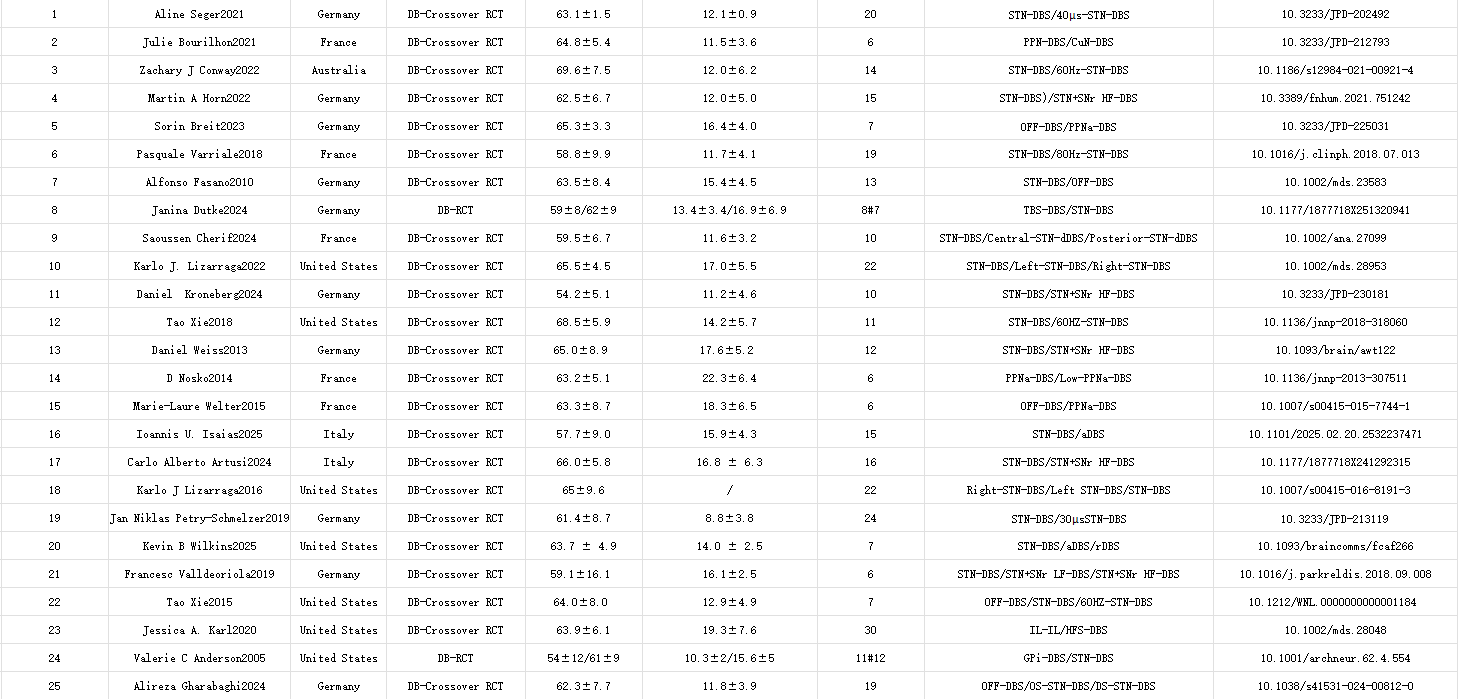
Table S1. Basic Information of All Included Literature

Table S2. Specific outcome data reported in the included studies.

| **Study** | **treatment** | **n** | MDS-UPDRSIII | mean | sd | FOG-Q | mean | sd | Gait Speed | **mean** | **sd** |
| --- | --- | --- | --- | --- | --- | --- | --- | --- | --- | --- | --- |
| 1 | OFF-DBS | 20 |  | 36.1 | 4.6 |  | 7.4 | 2.4 |  | 93.8 | 7.2 |
| 1 | STN-DBS | 20 |  | 25.6 | 3.4 |  | 4.4 | 1.3 |  | 107.2 | 5.1 |
| 1 | spDBS | 20 |  | 26.2 | 3.3 |  | 4 | 1.2 |  | 109.5 | 3.9 |
| 2 | PPN-DBS | 6 |  | 34 | 6.9 |  | 38.3 | 3.3 |  | \ | \ |
| 2 | CuN-DBS | 6 |  | 37 | 11.7 |  | 40.5 | 6.1 |  | \ | \ |
| 3 | STN-DBS | 14 |  | 32.7 | 10.7 |  | \ | \ |  | 108 | 15 |
| 3 | 60HZ-STN-DBS | 10 |  | 30.9 | 9.8 |  | \ | \ |  | 108 | 19 |
| 4 | OFF-DBS | 11 |  | 34.6 | 9.5 |  | \ | \ |  | 112.1 | 18.8 |
| 4 | STN-DBS | 11 |  | 19.6 | 8 |  | \ | \ |  | 114.5 | 18.9 |
| 4 | STN + SNr HF-DBS | 11 |  | 22.1 | 13 |  | \ | \ |  | 119.5 | 22.3 |
| 5 | OFF-DBS | 7 |  | 51 | 9.3 |  | 19.6 | 2.4 |  | \ | \ |
| 5 | PPNa-DBS | 7 |  | 46.4 | 13.1 |  | 18.1 | 4 |  | \ | \ |
| 6 | STN-DBS | 19 |  | 17.2 | 8.9 |  | 17.5 | 12.2 |  | \ | \ |
| 6 | 80HZ-STN-DBS | 19 |  | 13.2 | 8.5 |  | 11.3 | 8.4 |  | \ | \ |
| 7 | OFF-DBS | 13 |  | \ | \ |  | \ | \ |  | 39 | 5 |
| 7 | STN-DBS | 13 |  | \ | \ |  | \ | \ |  | 50 | 3 |
| 8 | STN-DBS | 7 |  | 28.4 | 9.9 |  | 26.6 | 9.8 |  | \ | \ |
| 8 | TBS-DBS | 8 |  | 27.9 | 8.1 |  | 23.1 | 10.3 |  | \ | \ |
| 9 | STN-DBS | 10 |  | 33.8 | 3.1 |  | \ | \ |  | 55.1 | 11.9 |
| 9 | Central-STN-dDBS | 10 |  | 34 | 3.1 |  | \ | \ |  | 47.1 | 17.3 |
| 9 | Posterior-STN-dDBS | 10 |  | 26.4 | 3.1 |  | \ | \ |  | 52.3 | 8.3 |
| 10 | STN-DBS | 22 |  | 48.9 | 10.6 |  | 18.8 | 14.9 |  | 94.1 | 8.6 |
| 10 | Right-STN-DBS | 22 |  | 49.2 | 15.7 |  | 13.3 | 6 |  | 87.2 | 4.8 |
| 10 | Left-STN-DBS | 22 |  | 53.2 | 16.1 |  | 14.7 | 4.6 |  | 92.6 | 4.8 |
| 11 | OFF-DBS | 10 |  | 36.6 | 9.5 |  | \ | \ |  | 58.9 | 25.4 |
| 11 | STN-DBS | 10 |  | 21.3 | 9.4 |  | \ | \ |  | 67.7 | 21 |
| 11 | STN+SNr-DBS | 10 |  | 21.7 | 9.6 |  | \ | \ |  | 67.1 | 24.8 |
| 12 | OFF-DBS | 11 |  | 31.4 | 10.1 |  | 7 | 4 |  | \ | \ |
| 12 | STN-DBS | 11 |  | 32.4 | 7.1 |  | 11.2 | 4.1 |  | \ | \ |
| 12 | 60HZ-STN-DBS | 11 |  | 27.1 | 9.1 |  | 5.9 | 4.4 |  | \ | \ |
| 13 | STN-DBS | 12 |  | 28.8 | 6 |  | 16.2 | 3.8 |  | \ | \ |
| 13 | STN+SNr HF-DBS | 12 |  | 29.8 | 5.5 |  | 14.5 | 4.9 |  | \ | \ |
| 14 | PPN-DBS | 6 |  | 29.1 | 13 |  | \ | \ |  | \ | \ |
| 14 | Low-PPNa-DBS | 6 |  | 23.9 | 13.1 |  | \ | \ |  | \ | \ |
| 15 | OFF-DBS | 6 |  | 46 | 8.7 |  | \ | \ |  | \ | \ |
| 15 | PPN-DBS | 6 |  | 20 | 5.2 |  | \ | \ |  | \ | \ |
| 16 | STN-DBS | 15 |  | 32.6 | 16.7 |  | \ | \ |  | \ | \ |
| 16 | aDBS | 15 |  | 31.5 | 17.7 |  | \ | \ |  | \ | \ |
| 17 | STN-DBS | 15 |  | 22 | 11.8 |  | 16.8 | 7.9 |  | \ | \ |
| 17 | STN + SNr HF-DBS | 15 |  | 18.6 | 11.6 |  | 16.6 | 9 |  | \ | \ |
| 17 | STN + SNr LF-DBS | 15 |  | 23.2 | 13.5 |  | 15.7 | 8.5 |  | \ | \ |
| 18 | STN-DBS | 22 |  | 24.1 | 2.8 |  | \ | \ |  | 95 | 5 |
| 18 | Right-STN-DBS | 22 |  | 35.6 | 3.2 |  | \ | \ |  | 92 | 5 |
| 18 | Left-STN-DBS | 22 |  | 38.4 | 3.5 |  | \ | \ |  | 90 | 5 |
| 19 | 60μsSTN-DBS | 24 |  | 16.9 | 6.5 |  | \ | \ |  | \ | \ |
| 19 | STN-DBS | 24 |  | 16.1 | 6.6 |  | \ | \ |  | \ | \ |
| 20 | aDBS | 7 |  | 19.7 | 7.4 |  | \ | \ |  | \ | \ |
| 20 | rDBS | 7 |  | 201 | 6.5 |  | \ | \ |  | \ | \ |
| 20 | STN-DBS | 7 |  | 19.6 | 6 |  | \ | \ |  | \ | \ |
| 21 | SNr LF-DBS | 6 |  | 27 | 6.7 |  | \ | \ |  | \ | \ |
| 21 | STN+SNr LF-DBS | 6 |  | 16.3 | 4.7 |  | \ | \ |  | \ | \ |
| 21 | STN-DBS | 6 |  | 27.3 | 6.7 |  | \ | \ |  | \ | \ |
| 22 | STN-DBS | 7 |  | 31.3 | 7.8 |  | 9.57 | 6.8 |  | \ | \ |
| 22 | 60HZ-STN-DBS | 7 |  | 15.4 | 7.7 |  | 2.29 | 4.42 |  | \ | \ |
| 23 | IL-IL-DBS | 20 |  | 28.89 | 10.3 |  | 14.5 | 9.5 |  | \ | \ |
| 23 | STN-DBS | 20 |  | 34.11 | 13.44 |  | 18.4 | 12 |  | \ | \ |
| 24 | GPi-DBS | 10 |  | 30 | 17 |  | \ | \ |  | \ | \ |
| 24 | STN-DBS | 10 |  | 27 | 11 |  | \ | \ |  | \ | \ |
| 25 | OFF-DBS | 19 |  | 39.6 | 12.9 |  | \ | \ |  | \ | \ |
| 25 | OS-STN-DBS | 19 |  | 30 | 13.6 |  | \ | \ |  | \ | \ |
| 25 | DS‑STN‑DBS | 19 |  | 30.7 | 10.9 |  | \ | \ |  | \ | \ |

Table S3. Search strategies for each database

Pubmed：

("Parkinson Disease"[Mesh] OR "Parkinson's Disease" OR Parkinsonism) AND ("Deep Brain Stimulation"[Mesh] OR "DBS" OR "adaptive DBS" OR "aDBS" OR "directional DBS"OR "closed-loop stimulation" OR "STN stimulation" OR "GPi stimulation" OR "VIM stimulation" OR"dual-target stimulation" OR "personalized DBS") AND ("Gait"[Mesh] OR "Gait Disorder" OR "Walking" OR "Locomotion") AND("Randomized Controlled Trial"[Publication Type] OR "RCT" OR "clinical trial")

Cochrane Library：

("Parkinson's Disease" OR Parkinsonism)AND("deep brain stimulation" OR DBS

OR "adaptive DBS" OR aDBSOR "directional DBS"OR "closed-loop stimulation"OR "STN stimulation"OR "GPi stimulation"OR "VIM stimulation"OR "dual-target stimulation"OR "personalized DBS")AND(gait OR "gait disorder" OR walking OR locomotion)

Embase：

('parkinson disease'/exp OR 'parkinson's disease':ti,ab OR parkinsonism:ti,ab) AND ('deep brain stimulation'/exp OR dbs:ti,ab OR 'adaptive dbs':ti,ab OR adbs:ti,ab OR 'directional dbs':ti,ab OR 'closed-loop stimulation':ti,ab OR 'stn stimulation':ti,ab OR 'gpi stimulation':ti,ab OR 'vim stimulation':ti,ab OR 'dual-target stimulation':ti,ab OR 'personalized dbs':ti,ab) AND ('gait'/exp OR 'gait disorder':ti,ab OR walking:ti,ab OR locomotion:ti,ab) AND ('randomized controlled trial'/exp OR rct:ti,ab OR 'clinical trial':ti,ab) AND [2000-2025]/py

CNKI：

主题 =("帕金森病"OR"帕金森氏病") AND ("脑深部电刺激"OR"DBS"OR"适应性DBS"OR"aDBS"OR"方向性 DBS"OR"闭环刺激"OR "STN 刺激"OR"GPi 刺激"OR"VIM 刺激"OR"双靶点刺激"OR"个体化DBS") AND ("步态"OR"步态障碍"OR"行走能力"OR"运动能力") AND ("随机对照试验"OR"RCT"OR"临床试验")

Table S4. Pairwise Comparisons under the Common-Effects Model (Outcome Indicator: ①)

| treat1 | treat2 | MD | 95% CI |
| --- | --- | --- | --- |
| OFF-DBS | spDBS | 9.6141 | [7.2789; 11.9493] |
| OFF-DBS | STN-DBS | 10.0579 | [7.9121; 12.2037] |
| spDBS | STN-DBS | 0.4438 | [-1.5816; 2.4692] |
| CuN-DBS | PPN-DBS | 3 | [-7.8685; 13.8685] |
| 60HZ-STN-DBS | STN-DBS | -5.6622 | [-9.9625; -1.3619] |
| OFF-DBS | STN-DBS | 10.0579 | [7.9121; 12.2037] |
| OFF-DBS | STN+SNr HF-DBS | 11.1226 | [7.4332; 14.8121] |
| STN-DBS | STN+SNr HF-DBS | 1.0647 | [-2.1682; 4.2976] |
| OFF-DBS | PPNa-DBS | 4.6 | [-7.3013; 16.5013] |
| 80HZ-STN-DBS | STN-DBS | -4 | [-9.5338; 1.5338] |
| STN-DBS | TBS-DBS | 0.5 | [-8.7353; 9.7353] |
| Central-STN-dDBS | STN-DBS | 0.2 | [-2.5172; 2.9172] |
| Posterior-STN-dDBS | STN-DBS | -7.4 | [-10.1172; -4.6828] |
| Central-STN-dDBS | Posterior-STN-dDBS | 7.6 | [4.8828; 10.3172] |
| Left-STN-DBS | STN-DBS | 13.747 | [11.9231; 15.5709] |
| Right-STN-DBS | STN-DBS | 10.925 | [9.1920; 12.6581] |
| Left-STN-DBS | Right-STN-DBS | 2.822 | [0.8830; 4.7609] |
| OFF-DBS | STN-DBS | 10.0579 | [7.9121; 12.2037] |
| OFF-DBS | STN+SNr HF-DBS | 11.1226 | [7.4332; 14.8121] |
| STN-DBS | STN+SNr HF-DBS | 1.0647 | [-2.1682; 4.2976] |
| 60HZ-STN-DBS | OFF-DBS | -15.7201 | [-20.3807; -11.0595] |
| OFF-DBS | STN-DBS | 10.0579 | [7.9121; 12.2037] |
| 60HZ-STN-DBS | STN-DBS | -5.6622 | [-9.9625; -1.3619] |
| STN-DBS | STN+SNr HF-DBS | 1.0647 | [-2.1682; 4.2976] |
| Low-PPNa-DBS | PPN-DBS | -5.2 | [-19.9673; 9.5673] |
| OFF-DBS | PPN-DBS | 26 | [17.8900; 34.1100] |
| aDBS | STN-DBS | -0.1967 | [-6.3199; 5.9265] |
| STN-DBS | STN+SNr HF-DBS | 1.0647 | [-2.1682; 4.2976] |
| STN-DBS | STN+SNr LF-DBS | 5.6386 | [0.5256; 10.7517] |
| STN+SNr HF-DBS | STN+SNr LF-DBS | 4.5739 | [-1.1127; 10.2605] |
| Left-STN-DBS | STN-DBS | 13.747 | [11.9231; 15.5709] |
| Right-STN-DBS | STN-DBS | 10.925 | [9.1920; 12.6581] |
| Left-STN-DBS | Right-STN-DBS | 2.822 | [0.8830; 4.7609] |
| 60μsSTN-DBS | STN-DBS | 0.8 | [-2.9061; 4.5061] |
| aDBS | rDBS | -0.579 | [-7.5614; 6.4034] |
| aDBS | STN-DBS | -0.1967 | [-6.3199; 5.9265] |
| rDBS | STN-DBS | 0.3823 | [-6.0212; 6.7858] |
| SNr LF-DBS | STN-DBS | 3.2932 | [-3.7752; 10.3616] |
| SNr LF-DBS | STN+SNr LF-DBS | 8.9318 | [2.5238; 15.3398] |
| STN-DBS | STN+SNr LF-DBS | 5.6386 | [0.5256; 10.7517] |
| 60HZ-STN-DBS | STN-DBS | -5.6622 | [-9.9625; -1.3619] |
| IL-IL-DBS | STN-DBS | -5.22 | [-12.6410; 2.2010] |
| GPi-DBS | STN-DBS | 3 | [-9.5499; 15.5499] |
| DS-STN-DBS | OFF-DBS | -8.9 | [-16.4938; -1.3062] |
| OFF-DBS | OS-STN-DBS | 9.6 | [1.1714; 18.0286] |
| DS-STN-DBS | OS-STN-DBS | 0.7 | [-7.1369; 8.5369] |

Table S5. Pairwise Comparisons under the Random-Effects Model (Outcome Indicator: ①)

| treat1 | treat2 | MD | 95% CI |
| --- | --- | --- | --- |
| OFF-DBS | spDBS | 9.2156 | -0.1592 to 18.5904 |
| OFF-DBS | STN-DBS | 9.1543 | 3.4594 to 14.8491 |
| spDBS | STN-DBS | -0.0613 | -9.4064 to 9.2838 |
| CuN-DBS | PPN-DBS | 3 | -11.8071 to 17.8071 |
| 60HZ-STN-DBS | STN-DBS | -5.5278 | -12.6081 to 1.5525 |
| OFF-DBS | STN+SNr HF-DBS | 11.1494 | 3.8884 to 18.4103 |
| STN-DBS | STN+SNr HF-DBS | 1.9951 | -3.9903 to 7.9805 |
| OFF-DBS | PPNa-DBS | 4.6 | -10.9809 to 20.1809 |
| 80HZ-STN-DBS | STN-DBS | -4 | -15.4781 to 7.4781 |
| STN-DBS | TBS-DBS | 0.5 | -13.1534 to 14.1534 |
| Central-STN-dDBS | STN-DBS | 0.2 | -10.2167 to 10.6167 |
| Posterior-STN-dDBS | STN-DBS | -7.4 | -17.8167 to 3.0167 |
| Central-STN-dDBS | Posterior-STN-dDBS | 7.6 | -2.8167 to 18.0167 |
| Left-STN-DBS | STN-DBS | 10.2856 | 2.2809 to 18.2903 |
| Right-STN-DBS | STN-DBS | 7.0422 | -0.9343 to 15.0186 |
| Left-STN-DBS | Right-STN-DBS | 3.2434 | -4.9770 to 11.4639 |
| Low-PPNa-DBS | PPN-DBS | -5.2 | -23.0661 to 12.6661 |
| OFF-DBS | PPN-DBS | 26 | 13.0811 to 38.9189 |
| aDBS | STN-DBS | -0.3486 | -10.0700 to 9.3727 |
| aDBS | rDBS | -0.6396 | -12.3982 to 11.1190 |
| rDBS | STN-DBS | 0.291 | -11.1903 to 11.7722 |
| SNr LF-DBS | STN-DBS | 3.3035 | -8.4086 to 15.0156 |
| SNr LF-DBS | STN+SNr LF-DBS | 7.7598 | -3.6306 to 19.1502 |
| STN-DBS | STN+SNr LF-DBS | 4.4563 | -4.1072 to 13.0198 |
| 60μsSTN-DBS | STN-DBS | 0.8 | -9.9173 to 11.5173 |
| IL-IL-DBS | STN-DBS | -5.22 | -17.7179 to 7.2779 |
| GPi-DBS | STN-DBS | 3 | -13.0818 to 19.0818 |
| DS-STN-DBS | OFF-DBS | -8.9 | -21.5012 to 3.7012 |
| OFF-DBS | OS-STN-DBS | 9.6 | -3.5212 to 22.7212 |
| DS-STN-DBS | OS-STN-DBS | 0.7 | -12.0492 to 13.4492 |

Table S6. Common-Effects Model: Treatment Effects vs OFF-DBS (Outcome Indicator: ①)

| Treatment | MD | 95% CI | p-value |
| --- | --- | --- | --- |
| 60HZ-STN-DBS | -15.7201 | -20.3807 to -11.0595 | <0.0001 |
| 60μsSTN-DBS | -9.2579 | -13.5403 to -4.9755 | <0.0001 |
| 80HZ-STN-DBS | -14.0579 | -19.9931 to -8.1227 | <0.0001 |
| aDBS | -10.2546 | -16.7429 to -3.7663 | 0.0020 |
| Central-STN-dDBS | -9.8579 | -13.3202 to -6.3956 | <0.0001 |
| CuN-DBS | -23 | -36.5609 to -9.4391 | 0.0009 |
| DS-STN-DBS | -8.9 | -16.4938 to -1.3062 | 0.0216 |
| GPi-DBS | -7.0579 | -19.7899 to 5.6741 | 0.2773 |
| IL-IL-DBS | -15.2779 | -23.0029 to -7.5529 | 0.0001 |
| Left-STN-DBS | 3.6891 | 0.8729 to 6.5053 | 0.0102 |
| Low-PPNa-DBS | -31.2 | -48.0477 to -14.3523 | 0.0003 |
| OS-STN-DBS | -9.6 | -18.0286 to -1.1714 | 0.0256 |
| Posterior-STN-dDBS | -17.4579 | -20.9202 to -13.9956 | <0.0001 |
| PPN-DBS | -26 | -34.1100 to -17.8900 | <0.0001 |
| PPNa-DBS | -4.6 | -16.5013 to 7.3013 | 0.4487 |
| rDBS | -9.6756 | -16.4290 to -2.9221 | 0.0050 |
| Right-STN-DBS | 0.8671 | -1.8911 to 3.6254 | 0.5378 |
| SNr LF-DBS | -6.7647 | -14.1382 to 0.6088 | 0.0722 |
| spDBS | -9.6141 | -11.9493 to -7.2789 | <0.0001 |
| STN-DBS | -10.0579 | -12.2037 to -7.9121 | <0.0001 |
| STN+SNr HF-DBS | -11.1226 | -14.8121 to -7.4332 | <0.0001 |
| STN+SNr LF-DBS | -15.6965 | -21.2150 to -10.1780 | <0.0001 |
| TBS-DBS | -10.5579 | -20.0392 to -1.0766 | 0.0291 |

Table S7. Pairwise Comparisons under the Common-Effects Model (Outcome Indicator: ②)

| treat1 | treat2 | SMD | 95%CI |
| --- | --- | --- | --- |
| OFF-DBS | spDBS | 1.2548 | [0.5992; 1.9104] |
| OFF-DBS | STN-DBS | 0.5956 | [0.0659; 1.1253] |
| spDBS | STN-DBS | -0.6592 | [-1.2576; -0.0608] |
| OFF-DBS | PPNa-DBS | 0.4548 | [-0.6063; 1.5159] |
| 80HZ-STN-DBS | STN-DBS | -0.592 | [-1.2416; 0.0577] |
| STN-DBS | TBS-DBS | 0.3475 | [-0.6745; 1.3695] |
| Left-STN-DBS | STN-DBS | -0.425 | [-1.0204; 0.1704] |
| Right-STN-DBS | STN-DBS | -0.5701 | [-1.1691; 0.0288] |
| Left-STN-DBS | Right-STN-DBS | 0.1451 | [-0.4463; 0.7366] |
| 60HZ-STN-DBS | OFF-DBS | -1.2353 | [-1.9381; -0.5324] |
| OFF-DBS | STN-DBS | 0.5956 | [0.0659; 1.1253] |
| 60HZ-STN-DBS | STN-DBS | -0.6397 | [-1.2987; 0.0193] |
| STN-DBS | STN+SNr HF-DBS | 0.1838 | [-0.3519; 0.7194] |
| STN-DBS | STN+SNr HF-DBS | 0.1838 | [-0.3519; 0.7194] |
| STN-DBS | STN+SNr LF-DBS | 0.2099 | [-0.4658; 0.8855] |
| STN+SNr HF-DBS | STN+SNr LF-DBS | 0.0261 | [-0.6495; 0.7017] |
| 60HZ-STN-DBS | STN-DBS | -0.6397 | [-1.2987; 0.0193] |
| IL-IL-DBS | STN-DBS | -0.3604 | [-0.9852; 0.2644] |

Table S8. Pairwise Comparisons under the Random-Effects Model (Outcome Indicator: ②)

| treat1 | treat2 | SMD | 95%-CI |
| --- | --- | --- | --- |
| OFF-DBS | spDBS | 1.2436 | [-0.9434; 3.4306] |
| OFF-DBS | STN-DBS | 0.3117 | [-1.3129; 1.9364] |
| spDBS | STN-DBS | -0.9319 | [-3.1053; 1.2416] |
| OFF-DBS | PPNa-DBS | 0.4548 | [-2.0248; 2.9344] |
| 80HZ-STN-DBS | STN-DBS | -0.592 | [-2.9253; 1.7414] |
| STN-DBS | TBS-DBS | 0.3475 | [-2.1156; 2.8106] |
| Left-STN-DBS | STN-DBS | -0.425 | [-2.7439; 1.8938] |
| Right-STN-DBS | STN-DBS | -0.5701 | [-2.8899; 1.7496] |
| Left-STN-DBS | Right-STN-DBS | 0.1451 | [-2.1727; 2.4630] |
| 60HZ-STN-DBS | OFF-DBS | -1.1839 | [-3.1479; 0.7801] |
| OFF-DBS | STN-DBS | 0.3117 | [-1.3129; 1.9364] |
| 60HZ-STN-DBS | STN-DBS | -0.8722 | [-2.5490; 0.8046] |
| STN-DBS | STN+SNr HF-DBS | 0.2034 | [-1.4705; 1.8773] |
| STN-DBS | STN+SNr HF-DBS | 0.2034 | [-1.4705; 1.8773] |
| STN-DBS | STN+SNr LF-DBS | 0.2196 | [-1.9831; 2.4224] |
| STN+SNr HF-DBS | STN+SNr LF-DBS | 0.0163 | [-2.1865; 2.2190] |
| 60HZ-STN-DBS | STN-DBS | -0.8722 | [-2.5490; 0.8046] |
| IL-IL-DBS | STN-DBS | -0.3604 | [-2.6869; 1.9662] |

Table S9. Pairwise Comparisons under the Common-Effects Model (Outcome Indicator: ③)

| Study | treat1 | treat2 | MD | 95% CI |
| --- | --- | --- | --- | --- |
| 1 | OFF-DBS | spDBS | -15.2003 | [-18.7068, -11.6937] |
| 1 | OFF-DBS | STN-DBS | -12.6496 | [-16.3427, -8.9565] |
| 1 | spDBS | STN-DBS | 2.5507 | [-0.2369, 5.3383] |
| 3 | 60HZ-STN-DBS | STN-DBS | 0 | [-14.1568, 14.1568] |
| 4 | OFF-DBS | STN-DBS | -12.6496 | [-16.3427, -8.9565] |
| 4 | OFF-DBS | STN+SNr HF-DBS | -11.6698 | [-23.6119, 0.2724] |
| 4 | STN-DBS | STN+SNr HF-DBS | 0.9798 | [-10.9199, 12.8795] |
| 9 | Central-STN-dDBS | STN-DBS | -8 | [-21.0142, 5.0142] |
| 9 | Posterior-STN-dDBS | STN-DBS | -2.8 | [-11.7924, 6.1924] |
| 9 | Central-STN-dDBS | Posterior-STN-dDBS | -5.2 | [-17.0926, 6.6926] |
| 10 | Left-STN-DBS | STN-DBS | -3.1355 | [-5.5015, -0.7695] |
| 10 | Right-STN-DBS | STN-DBS | -4.9865 | [-7.3525, -2.6204] |
| 10 | Left-STN-DBS | Right-STN-DBS | 1.851 | [-0.1953, 3.8972] |
| 11 | OFF-DBS | STN-DBS | -12.6496 | [-16.3427, -8.9565] |
| 11 | OFF-DBS | STN+SNr HF-DBS | -11.6698 | [-23.6119, 0.2724] |
| 11 | STN-DBS | STN+SNr HF-DBS | 0.9798 | [-10.9199, 12.8795] |
| 18 | Left-STN-DBS | STN-DBS | -3.1355 | [-5.5015, -0.7695] |
| 18 | Right-STN-DBS | STN-DBS | -4.9865 | [-7.3525, -2.6204] |
| 18 | Left-STN-DBS | Right-STN-DBS | 1.851 | [-0.1953, 3.8972] |

Table S10. Pairwise Comparisons under the Random-Effects Model (Outcome Indicator: ③)

| Study | treat1 | treat2 | MD | 95% CI |
| --- | --- | --- | --- | --- |
| 1 | OFF-DBS | spDBS | -14.5369 | [-21.9425, -7.1313] |
| 1 | OFF-DBS | STN-DBS | -11.2475 | [-18.0168, -4.4782] |
| 1 | spDBS | STN-DBS | 3.2894 | [-3.8622, 10.4411] |
| 3 | 60HZ-STN-DBS | STN-DBS | 0 | [-15.7148, 15.7148] |
| 4 | OFF-DBS | STN-DBS | -11.2475 | [-18.0168, -4.4782] |
| 4 | OFF-DBS | STN+SNr HF-DBS | -10.889 | [-23.9184, 2.1404] |
| 4 | STN-DBS | STN+SNr HF-DBS | 0.3585 | [-12.5497, 13.2667] |
| 9 | Central-STN-dDBS | STN-DBS | -8 | [-22.6939, 6.6939] |
| 9 | Posterior-STN-dDBS | STN-DBS | -2.8 | [-14.0873, 8.4873] |
| 9 | Central-STN-dDBS | Posterior-STN-dDBS | -5.2 | [-18.9104, 8.5104] |
| 10 | Left-STN-DBS | STN-DBS | -3.2293 | [-8.6602, 2.2016] |
| 10 | Right-STN-DBS | STN-DBS | -4.9523 | [-10.3832, 0.4785] |
| 10 | Left-STN-DBS | Right-STN-DBS | 1.7231 | [-3.5175, 6.9636] |
| 11 | OFF-DBS | STN-DBS | -11.2475 | [-18.0168, -4.4782] |
| 11 | OFF-DBS | STN+SNr HF-DBS | -10.889 | [-23.9184, 2.1404] |
| 11 | STN-DBS | STN+SNr HF-DBS | 0.3585 | [-12.5497, 13.2667] |
| 18 | Left-STN-DBS | STN-DBS | -3.2293 | [-8.6602, 2.2016] |
| 18 | Right-STN-DBS | STN-DBS | -4.9523 | [-10.3832, 0.4785] |
| 18 | Left-STN-DBS | Right-STN-DBS | 1.7231 | [-3.5175, 6.9636] |

Table S11. Common-Effects Model: Treatment Effects vs OFF-DBS (Outcome Indicator: ③)

| Treatment | MD | 95% CI | z | p-value |
| --- | --- | --- | --- | --- |
| 60HZ-STN-DBS | 11.2475 | [-5.8632, 28.3582] | 1.29 | 0.1976 |
| Central-STN-dDBS | 3.2475 | [-12.9307, 19.4256] | 0.39 | 0.694 |
| Left-STN-DBS | 8.0182 | [-0.6604, 16.6968] | 1.81 | 0.0702 |
| Posterior-STN-dDBS | 8.4475 | [-4.7140, 21.6090] | 1.26 | 0.2084 |
| Right-STN-DBS | 6.2951 | [-2.3834, 14.9737] | 1.42 | 0.1551 |
| spDBS | 14.5369 | [7.1313, 21.9425] | 3.85 | 0.0001 |
| STN-DBS | 11.2475 | [4.4782, 18.0168] | 3.26 | 0.0011 |
| STN+SNr HF-DBS | 10.889 | [-2.1404, 23.9184] | 1.64 | 0.1014 |

Table S12.The number of studies and participants corresponding to each stimulation

| Stimulation Modality | Number of Studies | Number of Participants |
| --- | --- | --- |
| STN-DBS | 19 | 272 |
| STN+SNr HF-DBS | 5 | 59 |
| Low Hz STN-DBS | 4 | 51 |
| PPNa-DBS | 3 | 19 |
| Low μs STN-DBS | 2 | 44 |
| aDBS | 2 | 22 |
| Left-STN-DBS | 2 | 44 |
| Right-STN-DBS | 2 | 44 |
| IL-IL | 1 | 30 |
| STN+SNr LF-DBS | 1 | 6 |
| PPN-DBS | 1 | 6 |
| TBS-DBS | 1 | 8 |
| Low-PPNa-DBS | 1 | 6 |
| rDBS | 1 | 7 |
| CuN-DBS | 1 | 6 |
| Central-STN-dDBS | 1 | 10 |
| Posterior-STN-dDBS | 1 | 10 |
| GPi-DBS | 1 | 11 |
| OS-STN-DBS | 1 | 19 |
| DS-STN-DBS | 1 | 19 |

Figure S1. Search results of various databases


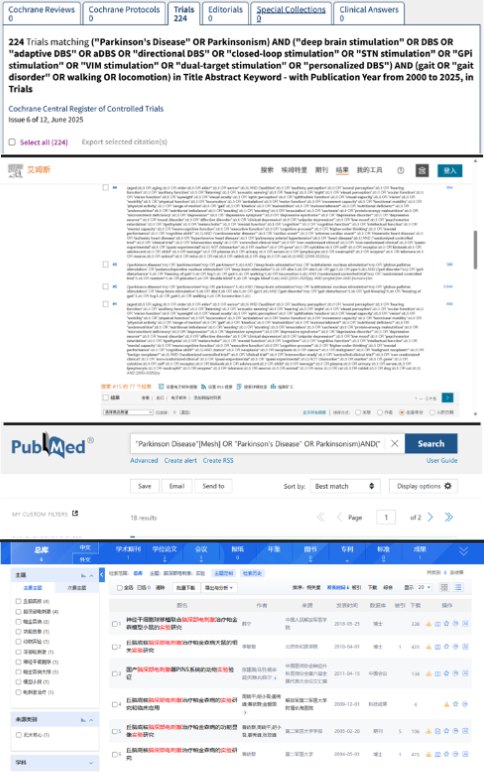


Figure S2. Risk assessment diagram of 25 articles.
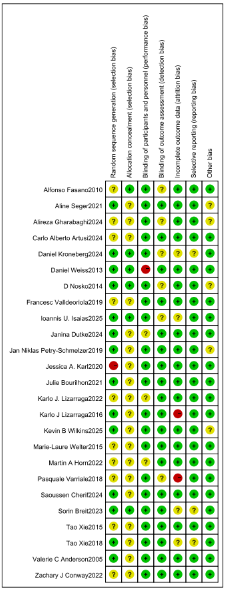


Figure S3. The published bias funnel plot：outcome indicators are A: ① / B: ② / C: ③
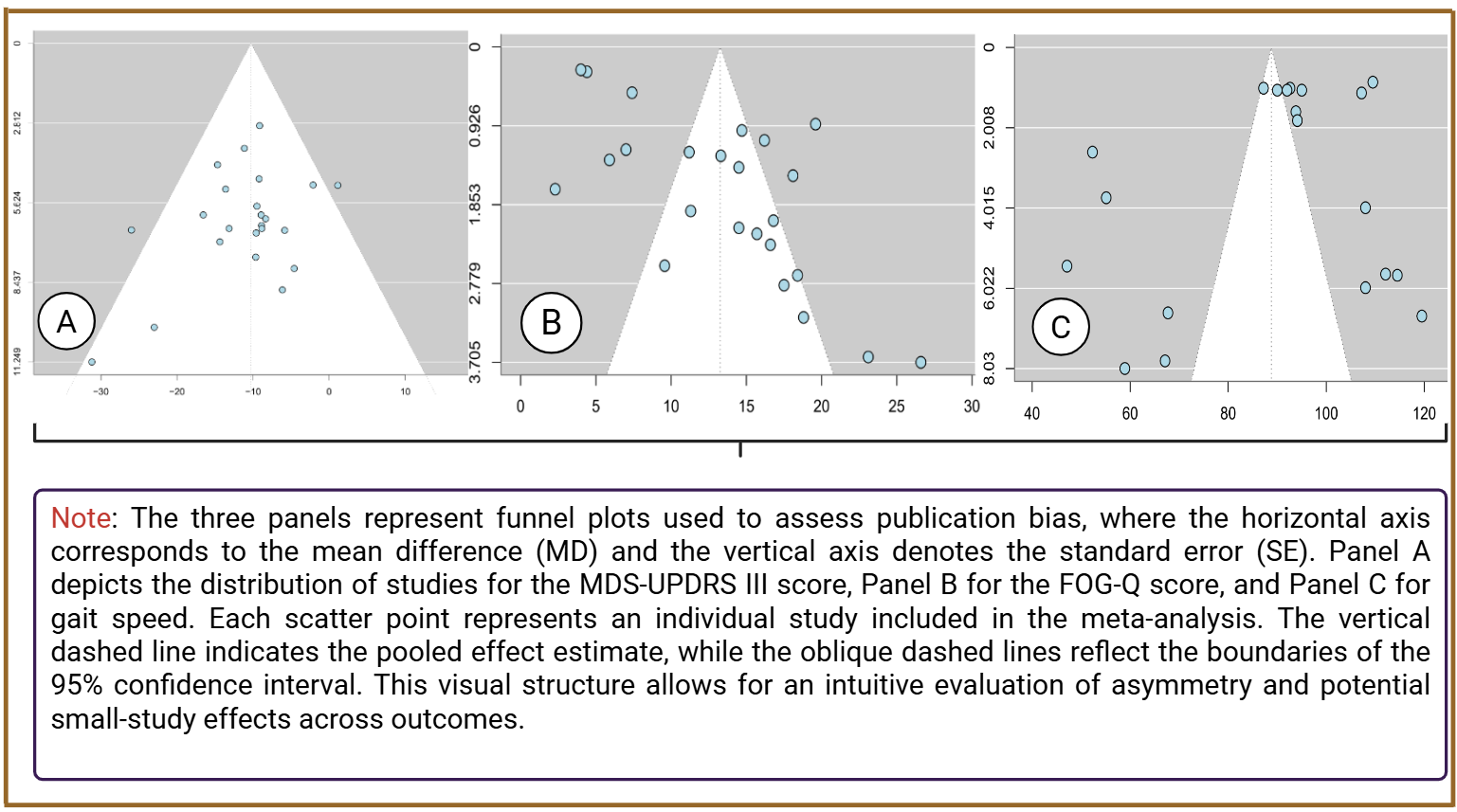


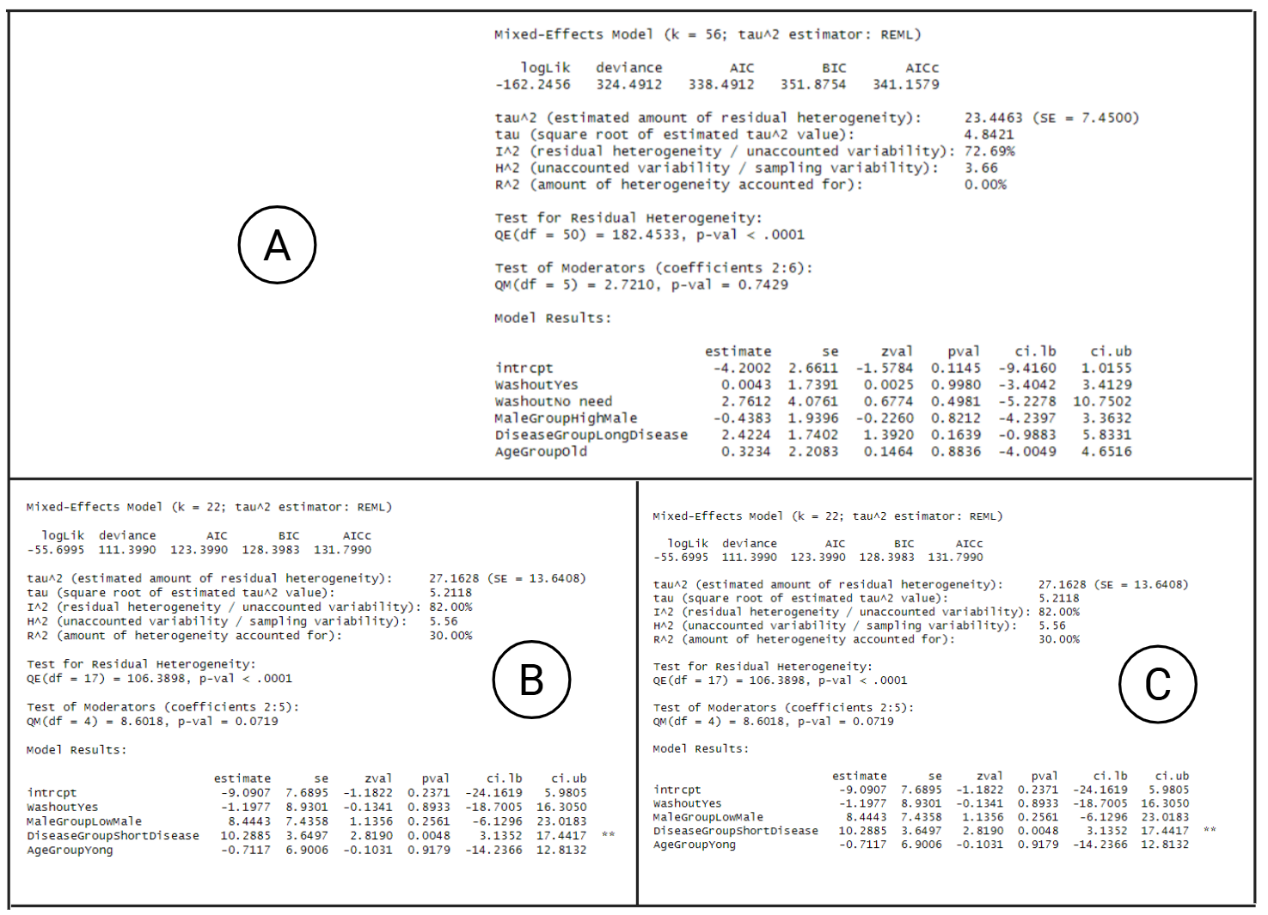
Figure S4. Meta-regression analysis results: Outcome indicators A:①/B:②/C:③

Note S1: In the protocol for our systematic review, registered prospectively on PROSPERO, we initially planned to search the CINAHL database as part of a comprehensive literature search strategy to ensure broad coverage of nursing, allied health, and biomedical literature relevant to the topic. This inclusion was intended to capture evidence from specialized sources that might not be fully represented in more general medical databases like MEDLINE or Embase. However, during the refinement of our search methods prior to commencing the formal literature search, we conducted a preliminary scoping review and pilot searches. These revealed that CINAHL yielded minimal additional relevant studies beyond those identified in our primary databases (e.g., PubMed, Embase, and Cochrane Library), with significant overlap and limited unique contributions specific to our research question. Furthermore, given the focus on network meta-analysis—which requires high-quality randomized controlled trials often indexed in core medical databases—we determined that excluding CINAHL would not compromise the comprehensiveness of our evidence synthesis while optimizing resource efficiency and reducing redundancy. This decision aligns with best practices in systematic reviews, where search strategies may be prospectively planned but refined based on feasibility and evidence yield, as long as changes are transparently documented to minimize bias. The final search was thus limited to the selected databases, and no studies from CINAHL were included in the network meta-analysis. This amendment was recorded in our review documentation to maintain transparency.

Note S2：Although our PROSPERO protocol specified a search cutoff date of 15 June 2025, a newly published study (17 July 2025) that fully met our eligibility criteria was identified during the revision process. To maintain the timeliness and completeness of this review, we included this study while keeping all predefined analytical methods unchanged. A sensitivity analysis was conducted to assess the impact of adding this study on the robustness of the pooled estimates.

Note S3：SMD was calculated using the Cohen’s d formula, which expresses the mean difference between two groups in units of pooled standard deviation. where $X$and $SD$ represent the mean and standard deviation of each group, and $n$ denotes the sample size. SMD expresses the effect size in units of standard deviation, facilitating comparison across studies even when different measurement scales are used. Positive SMD values indicate better outcomes in the treatment group, while negative values indicate worse outcomes. This approach was applied to all four continuous outcome measures in the current analysis. The small-sample bias has been corrected using SMD, yielding Hedges’ g.

$$\text{SMD}=\frac{X_{\text{treatment}}-X_{\text{control}}}{SD_{\text{pooled}}},SD_{\text{pooled}}=\sqrt{\frac{(n_{t}-1)SD_{t}^{2}+(n_{c}-1)SD_{c}^{2}}{n_{t}+n_{c}-2}}$$

**Appendix References:**

1. Hausdorff, J. M., Rios, D. A., & Edelberg, H. K. (2001). Gait variability and fall risk in community-living older adults: A 1-year prospective study. Archives of Physical Medicine and Rehabilitation, 82(8), 1050–1056. https://doi.org/10.1053/apmr.2001.24893

2. Yang, Y. R., Cheng, S. J., Lee, Y. J., Liu, Y. C., & Wang, R. Y. (2019). Cognitive and motor dual task gait training exerted specific training effects on dual task gait performance in individuals with Parkinson's disease: A randomized controlled pilot study. PLoS ONE, 14(6), e0218180. https://doi.org/10.1371/journal.pone.0218180

3. Gallardo-Gómez, D., Richardson, R., & Dwan, K. (2024). Standardized mean differences in meta-analysis: A tutorial. Cochrane Evidence Synthesis Methods, 2(3), e12047. https://doi.org/10.1002/cesm.12047

4. Hansen, C., Steinmetz, H., & Block, J. (2022). How to conduct a meta-analysis in eight steps: A practical guide. Management Review Quarterly, 72, 1–19. https://doi.org/10.1007/s11301-021-00247-4

5. Balduzzi, S., Rücker, G., Nikolakopoulou, A., Papakonstantinou, T., Salanti, G., Efthimiou, O., & Schwarzer, G. (2023). netmeta: An R package for network meta-analysis using frequentist methods. Journal of Statistical Software, 106(2), 1–40. https://doi.org/10.18637/jss.v106.i02

6. Borenstein, M., & Higgins, J. P. T. (2013). Meta-analysis and subgroups. Prevention Science, 14(2), 134–143. https://doi.org/10.1007/s11121-013-0377-7

7.Colditz, G. A. (2010). Overview of the epidemiology methods and applications: Strengths and limitations of observational study designs. Critical Reviews in Food Science and Nutrition, 50(Suppl 1), 10–12. https://doi.org/10.1080/10408398.2010.526838
